# Electrochemical Reduction of N_2_O with a Molecular Copper Catalyst

**DOI:** 10.1021/acscatal.3c02658

**Published:** 2023-09-14

**Authors:** Jorge L. Martinez, Joseph E. Schneider, Sophie W. Anferov, John S. Anderson

**Affiliations:** Department of Chemistry, University of Chicago, Chicago, Illinois 60637, United States

**Keywords:** N_2_O reduction, N_2_O reductase, homogeneous catalysis, electrocatalysis, redox-active ligand, denitrification

## Abstract

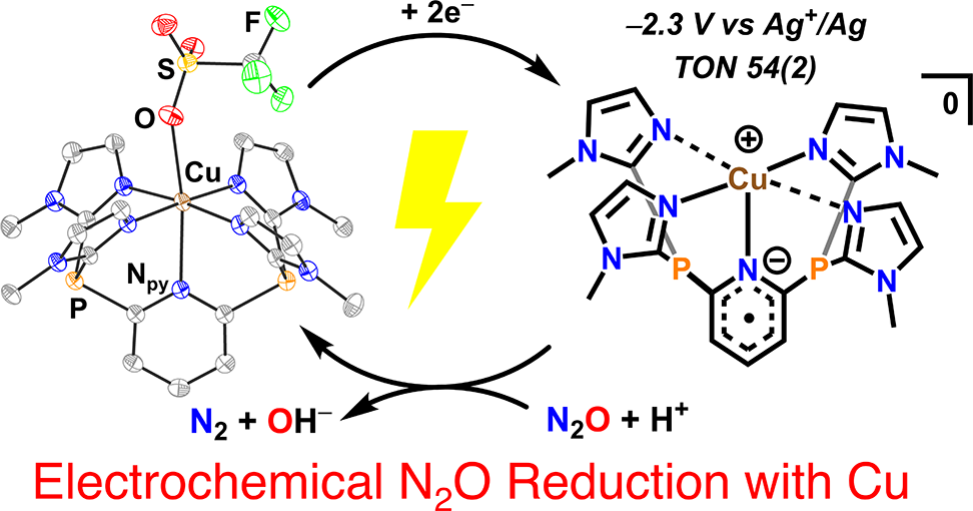

Deoxygenation of nitrous oxide (N_2_O) has significant environmental implications, as it is not only a potent greenhouse gas but is also the main substance responsible for the depletion of ozone in the stratosphere. This has spurred significant interest in molecular complexes that mediate N_2_O deoxygenation. Natural N_2_O reduction occurs via a Cu cofactor, but there is a notable dearth of synthetic molecular Cu catalysts for this process. In this work, we report a selective molecular Cu catalyst for the electrochemical reduction of N_2_O to N_2_ using H_2_O as the proton source. Cyclic voltammograms show that increasing the H_2_O concentration facilitates the deoxygenation of N_2_O, and control experiments with a Zn(II) analogue verify an essential role for Cu. Theory and spectroscopy support metal–ligand cooperative catalysis between Cu(I) and a reduced tetraimidazolyl-substituted radical pyridine ligand (MeIm_4_P_2_Py = 2,6-(bis(bis-2-*N*-methylimidazolyl)phosphino)pyridine), which can be observed by Electron Paramagnetic Resonance (EPR) spectroscopy. Comparison with biological processes suggests a common theme of supporting electron transfer moieties in enabling Cu-mediated N_2_O reduction.

## Introduction

Nitrous oxide (N_2_O) is the leading ozone-depleting substance of this century, rivaling many hydrochlorofluorocarbons (HCFCs).^1^ N_2_O is also a potent greenhouse gas with a long atmospheric lifetime (>100 years) and is 10× and 300× more warming by mass than CH_4_ and CO_2_, respectively.^2,3^ Thus, decomposition of N_2_O is important in order to mitigate its effect on climate. Of possible decomposition routes, the reduction of N_2_O to N_2_ and H_2_O is attractive due to the formation of benign byproducts. However, deoxygenation remains difficult, as even thermal decomposition is limited by a large kinetic barrier (Δ*G*^‡^ = 59 kcal/mol in the gas phase) despite being thermodynamically favorable (Δ*G* = −81 kcal/mol).^4,5^ The poor σ-donating and π-accepting properties of N_2_O lead to sluggish binding kinetics with transition metals, which also limits their chemistry with N_2_O.^6,7^ This problem is reflected by the small number of N_2_O adducts that have been characterized structurally and/or spectroscopically.^8−14^

**Figure 1:**
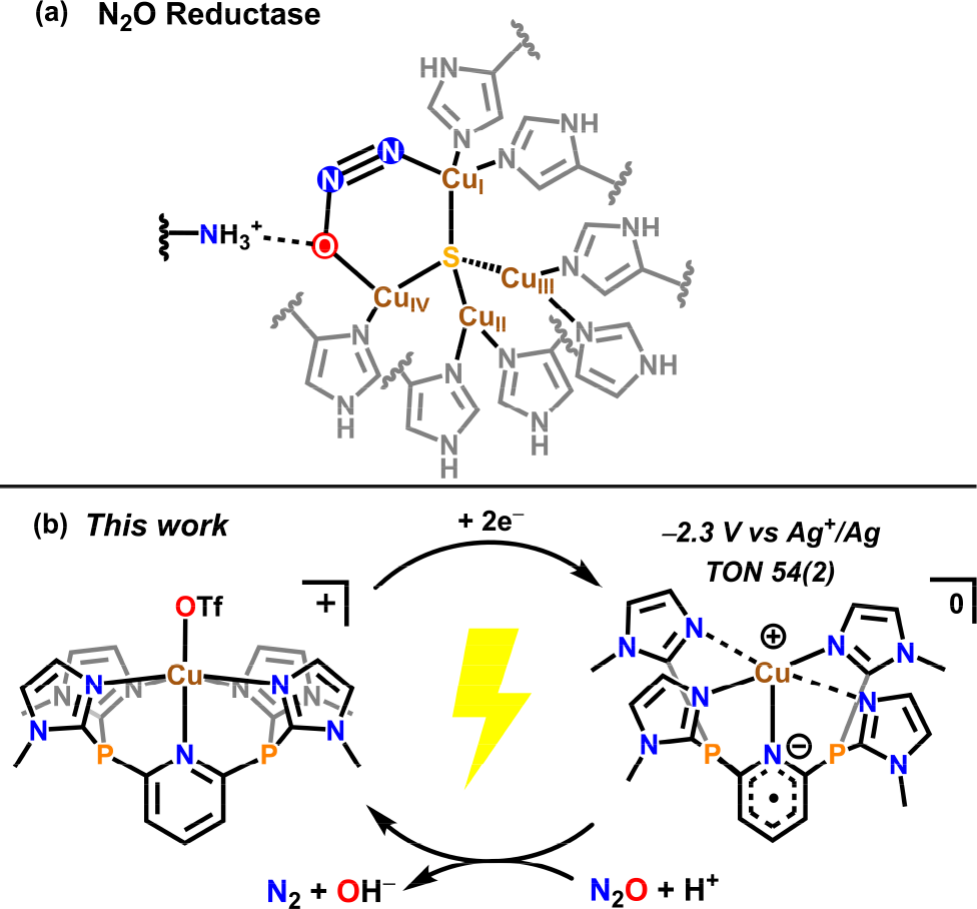
(a) Proposed binding of N_2_O in the 4Cu^I^:S active site of N_2_O reductase and (b) electrochemical N_2_O reduction catalysis in this work.

Approximately two-thirds of anthropogenic N_2_O is derived from excess Haber–Bosch nitrogen (i.e., fertilizers) through incomplete bacterial denitrification.^15−17^ Some denitrification bacteria contain the gene for nitrous oxide reductase (N_2_OR), the only known enzyme capable of reducing N_2_O as its natural substrate to N_2_ and H_2_O through a 2e^–^/2H^+^ process.^18^ N_2_OR is a Cu-based metalloenzyme consisting of two Cu clusters: a binuclear copper site responsible for electron transfer, Cu_A_, and a tetranuclear μ_4_-sulfido bridged cluster, Cu_Z_*, which is the active site for N_2_O binding and reduction.^19,20^ Computational and spectroscopic studies suggest that N_2_OR overcomes the high kinetic barrier for N–O cleavage by binding N_2_O in a μ-1,3 fashion assisted by H-bonding from the secondary coordination sphere (Figure 1a),^21,22^ although there is some controversy over this mechanistic proposal.^23−25^

Despite the importance of Cu in biological N_2_O reduction, and elegant synthetic work aimed at isolating structurally and chemically faithful active site mimics,^26−33^ molecular Cu catalysts for the reduction of N_2_O to N_2_ and H_2_O/OH^–^ are not known. Nevertheless, stoichiometric reactions with di- and trinuclear Cu species suggest that tetranuclear copper sites are not required for N_2_O reduction.^34^ Favorable binding of N_2_O to Cu(I) suggests that even monometallic systems should also be considered.^8,35^

Despite the lack of synthetic Cu-based N_2_O reduction catalysts, homogeneous thermal hydrogenation of N_2_O to N_2_ and H_2_O has been reported with second and third row transition metal catalysts, namely, Ru, Rh, Pt, and Ir.^36−39^ Mixtures of N_2_O and H_2_ are potentially explosive,^40,41^ and this has also spurred interest in electrochemical reduction of N_2_O as an effective route.^42^ Still, while several exciting examples of molecular N_2_O electrocatalysts have recently been reported, no examples of Cu-based catalysts have been shown despite Cu’s biological relevance.^43−48^

These considerations motivated us to investigate the synthesis of a new N_2_O reduction electrocatalyst based on Cu. Herein we report the first molecular Cu complex capable of electrocatalytically reducing N_2_O in MeCN using water as a proton source. Electrochemical studies show that this catalyst operates with excellent selectivity for N_2_O reduction vs hydrogen evolution. Mechanistic analysis suggests that ligand redox noninnocence plays an important role, as verified by both computations and spectroscopy, and this observation may suggest a more general need for additional electron storing ligands/metals in molecular N_2_O reduction catalysis.

## Results and Discussion

### Catalyst Synthesis and Characterization

**Scheme 1:**
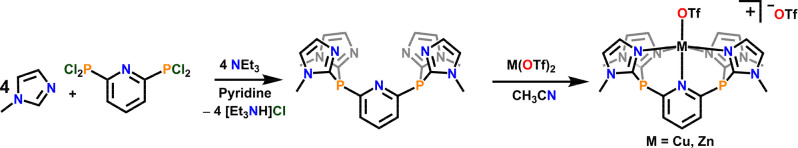
Complex Synthesis

It has previously been shown that some organic radicals are competent outer-sphere redox catalysts for the electrochemical reduction of N_2_O in MeCN,^47^ and we were particularly inspired by catalysis using 4-cyanopyridine.^49^ We were interested in investigating a pyridine donor that could be used as an effective electron shuttle for Cu-catalyzed N_2_O reduction (Figure 1b). Since demetalation of Cu(I) is a common deactivation pathway in Cu_Z_* synthetic models, we rationalized that polydentate chelates with strong N donors could minimize the loss of this labile metal.^30,33^ We previously reported a Co(II) compound supported by a tip(Me) ligand (tip(Me) = 2,6-(bis(bis-2-*N*-methylimidazolyl)-hydroxymethyl)pyridine) for electrocatalytic water oxidation featuring strongly donating imidazole arms.^50^ We hypothesized that strongly donating imidazole arms would chelate the metal more strongly versus other N5 polypyridyl ligands such as PY5Me_2_ (PY5Me_2_ = 2,6-bis(1,1-bis(2-pyridyl)ethyl)pyridine).^51−54^ We aimed to further redesign this ligand to remove the acidic hydroxyl groups and to increase synthetic ease by replacing the carbon bridgeheads with P atoms. This new tetraimidazolyl-substituted pyridine ligand, MeIm_4_P_2_Py (MeIm_4_P_2_Py = 2,6-(bis(bis-2-*N*-methylimidazolyl)phosphino)pyridine, **1**), was synthesized by adapting a previously described one-pot reaction between a dichlorophosphine and an *N*-alkyl imidazole (Scheme 1).^55^ Addition of 2,6-(Cl_2_P)Py to a mixture of *N*-methylimidazole and NEt_3_ in pyridine at room temperature affords **1** in a good yield after workup (60%).

**Figure 2:**
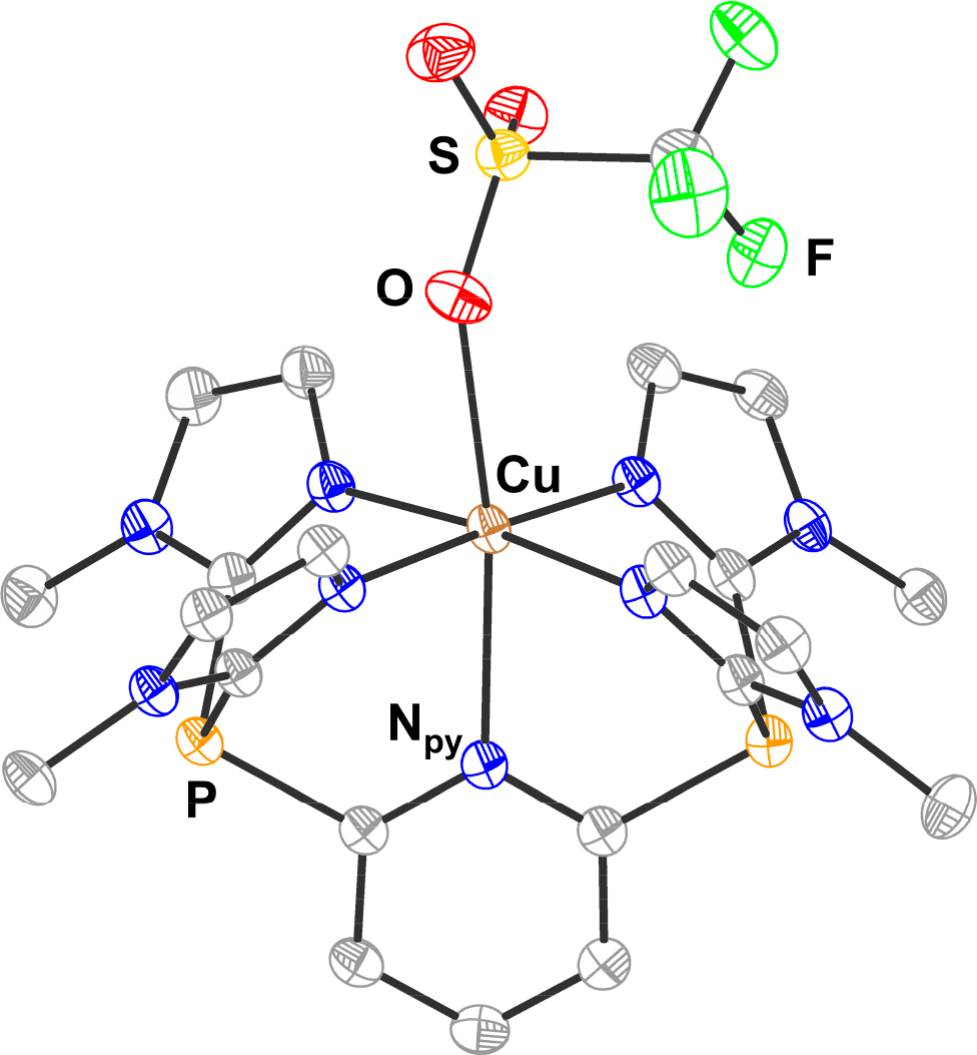
Solid-state structure of **1-Cu**. Ellipsoids are shown at 50% probability, and hydrogen atoms have been omitted for clarity. C is shown in gray, N is shown in blue, O is shown in red, F is shown in green, P is shown in orange, S is shown in yellow, and Cu is shown in brown. Selected bond lengths (Å) and angles (°) are as follows: Cu–N_Im_ 2.048(2), 2.013(2), 2.028(2), and 1.997(2); Co–N_Py_ 2.432(2); Co–O 2.487(2); O–Cu–N_Py_ 170.81(7); N5–Cu–N7 87.35(8); and N7–Cu–N3 92.67(8).

Metalation was carried out by the addition of Cu(OTf)_2_ to a slurry of sparingly soluble **1** in MeCN, affording [(MeIm_4_P_2_Py)Cu(OTf)][OTf] (**1-Cu**) as an air-stable blue powder in excellent yield after recrystallization (82%). Crystals suitable for single-crystal X-ray diffraction (SXRD) were obtained by the slow vapor diffusion of Et_2_O into MeCN at room temperature. The solid-state structure of **1-Cu** (Figure 2) reveals the pentadentate coordination of **1** to Cu with one inner sphere triflate ion. This six-coordinate species contains notably long axial bonds (Cu–O = 2.487(2) Å, Cu–N_Py_ = 2.432(2) Å) consistent with a Jahn–Teller distortion in tetragonally elongated Cu(II). Imidazole bond distances (⟨Cu–N_Im_⟩ = 2.022(2) Å) are slightly shorter than the nonelongated copper-pyridine distances in the analogous six-coordinate [(PY5Me_2_)Cu(MeCN)]^2+^ complex (⟨Cu–N_Py_⟩ = 2.044(4) Å).^56^ The metal center lies only 0.085 Å from the plane defined by the four imidazole N atoms. This is in contrast to our previously reported [Co(tip(Me))(CH_3_CN)][OTf]_2_ complex, which shows the metal “puckered” 0.213 Å away from the N_Im_ plane.^50^ This main structural difference between **1** and tip(Me) can be attributed to the larger size of phosphorus compared to carbon.

The frozen solution EPR spectrum of **1-Cu** (15 K) is consistent with a Cu(II) *S* = 1/2 assignment (Figure S39). Paramagnetically shifted resonances in the ^1^H NMR spectrum of **1-Cu** also support this d^9^ assignment (Figure S7), and Evan’s method shows an expected solution moment of 1.71(4) μ_B_. The number of resonances in the ^1^H NMR spectrum is also in agreement with the solid-state *C*_2*v*_ symmetry. A sharp singlet in the ^19^F{^1^H} NMR spectrum that is slightly shifted from free triflate suggests weakly ion paired triflate counteranions in CD_3_CN solution. Rapid ligand exchange between outer sphere triflate and MeCN is possible, if not likely, in solution. Additionally, a very broad resonance can be observed in the ^31^P{^1^H} NMR spectrum of **1-Cu** (δ = −48 ppm) in concentrated samples, corresponding to the bridging P atoms of the ligand with a chemical shift similar to that of **1** (δ = −45.2 ppm).

### Electrochemical Characterization

Cyclic voltammetry (CV) of **1-Cu** in MeCN revealed three reduction processes. The first quasi-reversible wave at −0.8 V vs Fc^+^/Fc corresponds to a metal-centered Cu(II)/Cu(I) redox couple with a large peak-to-peak separation (Δ*E*_p_ = 200 mV at 100 mV s^–1^) . A similarly large Δ*E*_p_ was observed in the previously reported PY5Me_2_ analog.^56^ Scan rate studies show an increase in Δ*E*_p_ of **1-Cu** with increasing scan rates, which indicates a changing coordination environment upon reduction from Cu(II) to Cu(I).^57^ The two remaining irreversible cathodic processes appear at *E*_p,c_ = −2.15 V and *E*_p,c_ = −2.75 V vs Fc^+^/Fc and are tentatively assigned as Cu(I)L/Cu(I)L^•–^ and Cu(I)L^•^/Cu(I)L^2–^ ligand-based reductions, respectively (L = **1**; Figure S15). These assignments are supported by the CV of **1** in MeCN, which shows that the substituted pyridine ligand can be reduced by 1 e^–^ in the absence of a metal (Figure S23).

To confirm the presence of ligand-based reductions in the CV of **1-Cu**, the control complex [(MeIm_4_P_2_Py)ZnOTf][OTf] (**1-Zn**) was synthesized following a similar procedure as its Cu(II) analog. An octahedral ligand field environment similar to that of **1-Cu** is observed in the SXRD structure of **1-Zn** (Figure S43). The average Zn–N_Im_ (2.118(1) Å) bond is slightly shorter than the average Zn–N_Py_ distance (2.153(4) Å) in its PY5Me_2_ analog, consistent with the stronger donor properties of the imidazole donors.^58^ Most notably, **1-Zn** also contains an unusually long axial Zn–N_Py_ bond (2.380(2) Å), which is again likely enforced by the longer bridging P–C bonds in ligand **1**.^59^

The CV of **1-Zn** in MeCN shows three cathodic processes (Figure S21), with the first irreversible wave assigned as the Zn(II)L^2+^/Zn(II)L^•+^ redox couple (*E*_p,c_ = −1.83 V). The remaining two, presumably ligand-based, irreversible reductions are at very negative potentials (*E*_p,c_ = −2.54 V and *E*_p,c_ = −2.73 V). Notably, the reduction of **1-Zn** is milder than that of the analogous [Zn(PY5Me_2_)(MeCN)]^2+^ complex, suggesting that the phosphine groups in **1** are more electron-withdrawing toward the apical pyridine ring.^60^ The ligand-based reduction potential in **1-Zn** is significantly shifted from those of **1-Cu** (*E*_p,c_ = −2.15 V) and the free ligand **1** (*E*_1/2_ = −2.68 V). These differences in potential can be attributed to the differences in charge between the three species. Regardless of these differences, all of these CV studies support the viability of a ligand-based reduction in this system.

### Electrocatalysis

**Figure 3:**
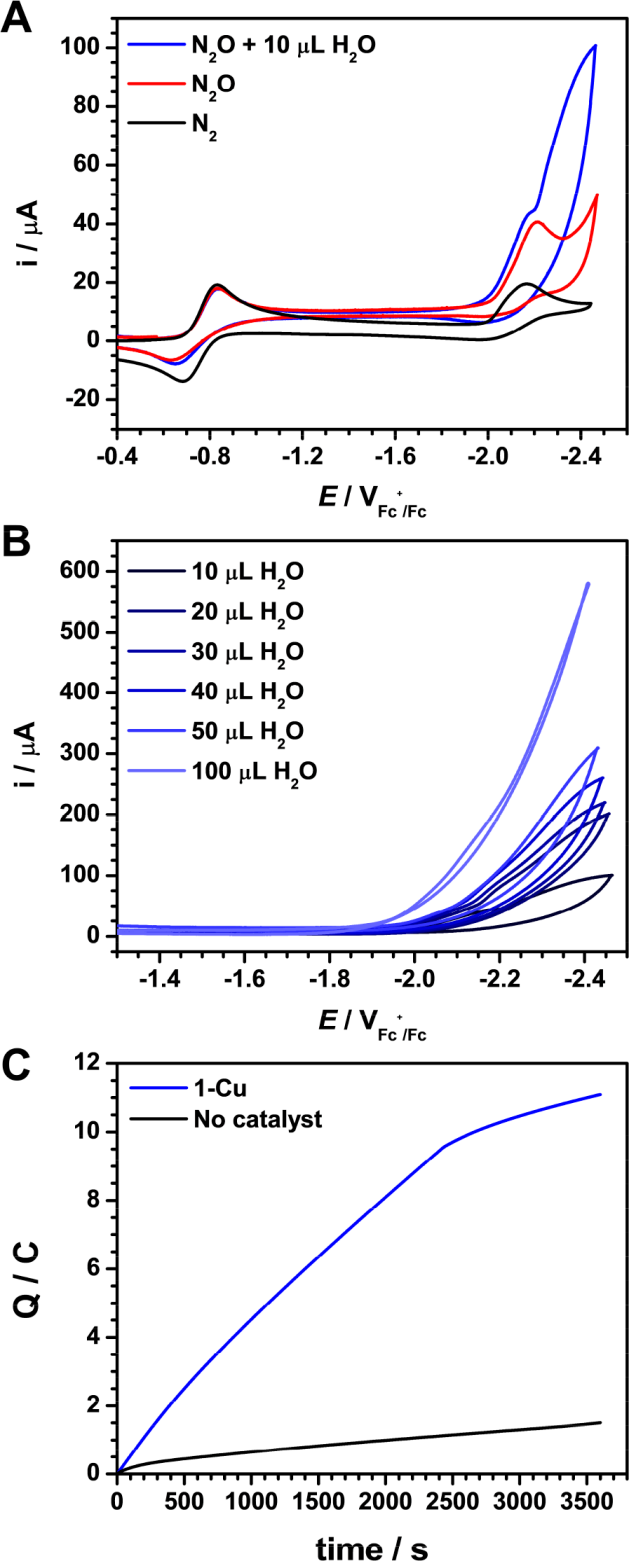
Cyclic voltammograms (scan rate 100 mV s^–1^) recorded using a 3 mm diameter glassy carbon working electrode in MeCN and 0.1 M N^*n*^Bu_4_PF_6_ supporting electrolyte for (a) **1-Cu** under 1 atm N_2_ (black), N_2_O (red), and N_2_O in the presence of 10 μL (0.1 M) of water (blue) and (b) **1-Cu** under 1 atm of N_2_O with increasing amounts of water: 10 (0.11 M), 20 (0.22 M), 30 (0.33 M), 40 (0.44 M), 50 (0.55 M), and 100 μL (1.11 M). (c) Controlled potential electrolysis at −2.3 V (vs Ag^+^/Ag) of a MeCN solution of 0.1 M N^*n*^Bu_4_PF_6_ supporting electrolyte with H_2_O (100 mM) under 1 atm N_2_O using a RVC working electrode. Charge passed as a function of time in the presence of catalyst (**1-Cu**, 1 mM) is shown in blue, and that in the absence of catalyst is shown in black.

In dry solvent and electrolyte, the first two reduction potentials in the CV of **1-Cu** under an atmosphere of N_2_O do not shift, suggesting that there is no binding of N_2_O prior to the reduction of the complex (Figure 3A). A catalytic current is observed upon the addition of water, and a slight anodic shift is observed with increasing equivalents (Figure 3B). The stoichiometry of this reaction is described in eq 1.
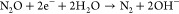
1Reduction of N_2_O occurs after the ligand is reduced, as can be seen by the inflection in the catalytic wave after the Cu(I)L/Cu(I)L^•–^ redox couple (Figure 3A, blue trace). The slight anodic shift in the inflection point with increasing water concentration could suggest that water facilitates binding to Cu(I) and promotes N–O bond cleavage. Evidence of significant water coordination to Cu was not found, as the reduction potentials of **1-Cu** remained unchanged with high concentrations of water in the absence of N_2_O (Figure S19). Similar electrochemical behavior is observed when the N^*n*^Bu_4_OTf supporting electrolyte is used instead of N^*n*^Bu_4_PF_6_, which excludes competitive OTf^–^ binding in solution (Figure S18). The CV of **1-Zn** in the presence of N_2_O shows that it is not a competent N_2_O reduction electrocatalyst, and we note that the reduction potential of ligand **1** is beyond the onset for the direct reduction of N_2_O with glassy carbon (see the SI for details). Both observations highlight the importance of Cu for electrocatalysis with this framework.

Controlled potential electrolysis (CPE) in the presence of 1 atm N_2_O and 100 mM H_2_O using a reticulated vitreous carbon (RVC) working electrode was performed at −2.3 V for 1 h to investigate the product selectivity of **1-Cu**. A linear increase of charge passed over time is seen up until ∼9 C is passed, after which charge consumption plateaus, indicating loss of activity (Figure 3C). This hypothesis is also supported by the slow drop in current over the course of electrolysis, which suggests catalyst degradation, as has been observed in related systems.^47^ We note that only a low amount of background activity by the RVC electrode was observed in the absence of **1-Cu** under identical conditions. Headspace analysis using TCD GC found N_2_ to be the only gaseous product, with no detectable H_2_. Additionally, only a small amount of H_2_ is produced in the absence of N_2_O under identical conditions (Figures S34 and S36), indicating that **1-Cu** is not competent for proton reduction under these conditions. An average turnover number (TON) of 54(2) was determined over the course of 1 h with a Faradaic efficiency of 83(8)% for N_2_. This nonquantitative Faradaic yield likely arises from decreasing activity over time due to catalyst degradation, presumably from competing reactions with water or the OH^–^ product^61^ as well as the limited stability of the reduced species. Further evidence of this is supported by the CV of **1-Cu**, which shows the instability of the reduced species in the presence of water at slower scan rates (Figure S19). Finally, CV of the solution after CPE using a glassy carbon plate (Figure S27) shows no electrochemical features, suggesting that the decomposition product(s) is electrochemically inactive. Indeed, a significant amount of free ligand, which is electrochemically inactive at the applied potential, was identified by ^1^H and ^31^P{^1^H} NMR spectroscopy after CPE (Figures S28 and S29).

### Mechanistic Investigations

The electrochemical activity of **1-Cu** prompted us to perform chemical and theoretical investigations of possible mechanistic steps. We hypothesized that any N_2_O reduction would necessarily proceed from an initially reduced congener of **1-Cu**. We therefore investigated the reactivity of **1-Cu** with the reducing agents. We initially tested catalytic chemical reduction of N_2_O using 0.1% Na/Hg as a reducing agent under conditions similar to those used in CPE experiments (see the SI). Although there is significant activity from Na/Hg and H_2_O in the absence of catalyst, the presence of 1 mol % **1-Cu** more than doubles the amount of N_2_ generated, with nearly all reducing agent consumed (Figure S33), supporting Cu-mediated catalysis. This observation also supports the use of Na/Hg as a surrogate for electrochemical reduction in mechanistic probes of **1-Cu**.

**Scheme 2:**
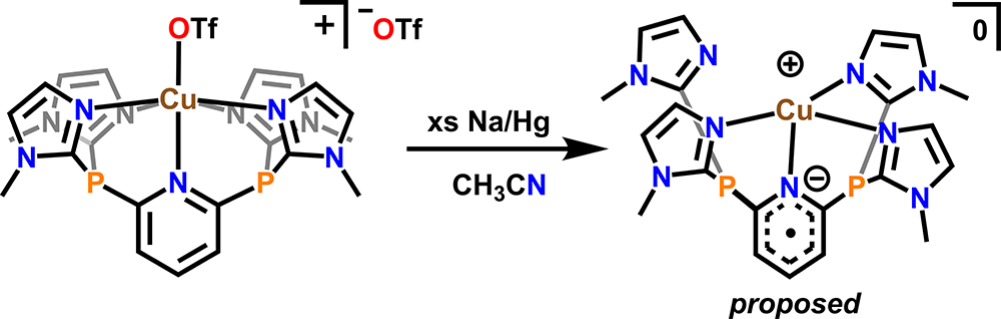
Reduction of **1-Cu** by 2e^–^ using Excess Na/Hg

We then attempted to obtain a more detailed characterization of the reduction products by chemically reducing **1-Cu** with Na/Hg in MeCN (Scheme 2). *In situ* monitoring of this reduction by UV–visible spectroscopy shows the disappearance of the initial d–d transitions in the spectrum, followed by a slow growth of two bands at approximately 410 and 680 nm over the course of 1 h (Figure S38). We propose that these absorption bands are related to the catalytically relevant species, since the Cu(I) intermediate is expected to have no signal in the visible region, consistent with our observations.

**Figure 4:**
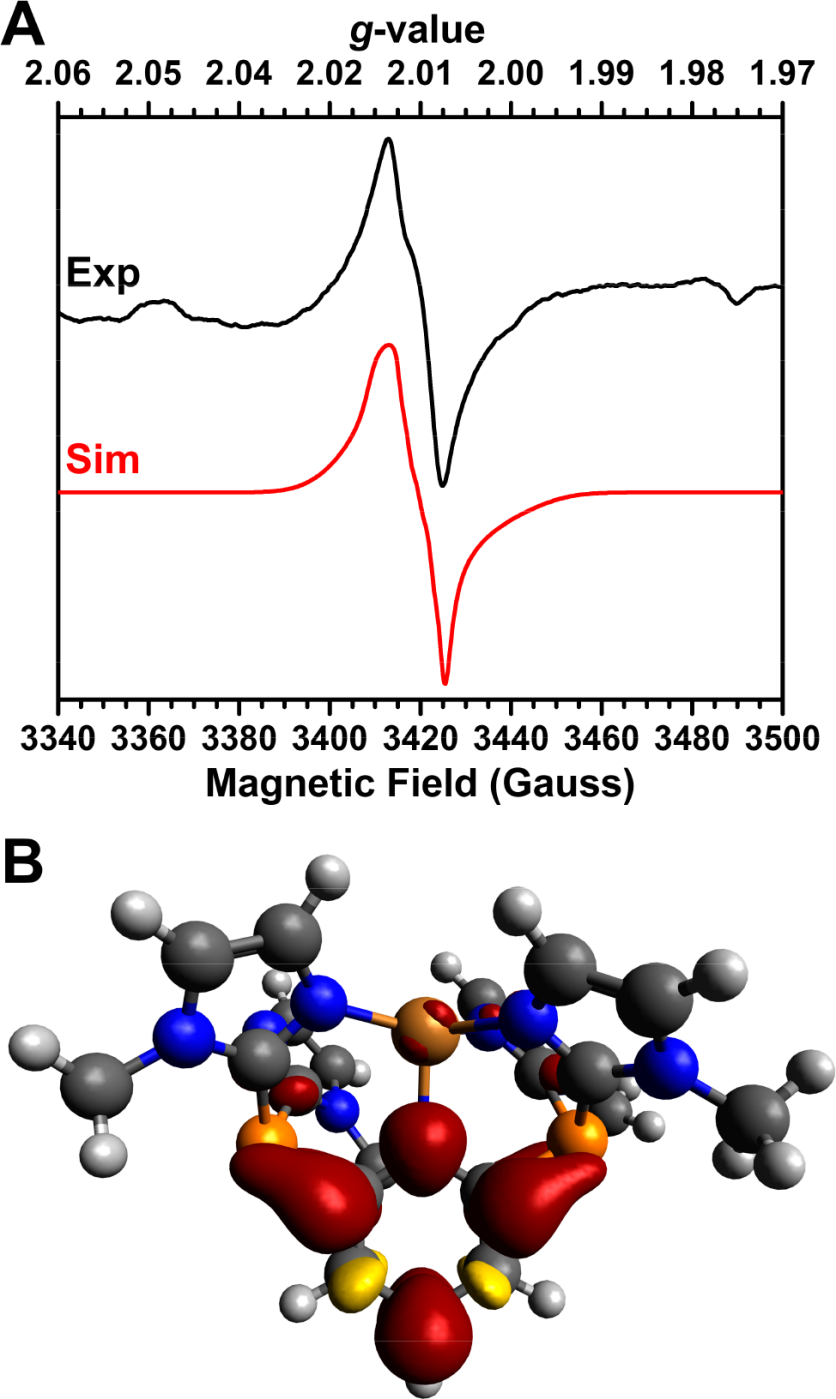
(A) EPR spectrum of the Na/Hg reduction of **1-Cu**. (B) Spin density plot of the 2e^–^ reduced model complex (isovalue of 0.002). EPR conditions are as follows: microwave frequency of 9.6304 GHz, microwave power of 0.2 mW, and modulation of 0.03 mT/100 kHz.

To test this hypothesis, characterization of this putative odd-electron-reduced species was attempted using EPR spectroscopy. An EPR signal consistent with an *S* = 1/2 complex distinct from the EPR signal of the Cu(II) starting complex was observed in a MeCN frozen solution (33 K) (Figure 4A). The frozen-solution EPR spectrum of the reduction of **1-Zn** (Figure S40) shows a similar but distinct signal that supports the viability of ligand-based radicals in this system. The signal from the reduction of **1-Cu** can be simulated with *g* = 2.010, 2.013, and 2.012, *A*(^63^Cu) = 12, 11, and 7 MHz, and *A*(^14^N) = 42, 3, and 2 MHz, consistent with a primarily pyridine centered radical. We note this signal is qualitatively similar to a previously reported 2,6-disubstituted pyridine radical (broad single line, peak-to-peak 120 G).^62^

We also performed DFT calculations on possible reduced complexes to gain insight into the catalytically relevant species (Figure S44). Geometry optimizations of a singly reduced Cu(I) intermediate predict a four-coordinate geometry with one ligand arm dissociated. This prediction is consistent with the structural changes inferred from the quasi-reversible redox couple in the CV of **1-Cu** at −0.8 V vs Fc^+^/Fc. Further reduction by an additional electron is ligand-based, as illustrated by the spin density, which is primarily on the pyridine donor with some delocalization onto the imidazole arms through the σ* of the P–C bond (Figure 4B). We have performed calculations of the EPR parameters of this complex, which match the values obtained from simulation of the experimental spectrum well. Namely, both DFT and simulation support moderate hyperfine coupling to N and Cu (Table S1).

It is important to note that there are multiple thermodynamically accessible isomers for these reduced species that may contribute to catalyst degradation, as has been reported in a similar N5 species for water reduction (see SI).^63^ Thus, the depicted geometry of this doubly reduced intermediate is only a model, and other coordination geometries and ligation environments (different solvates, for example) are possible if not likely. We hypothesize that this reduced species then rapidly reacts with N_2_O and water to generate N_2_ and OH^–^, consistent with the catalytic onset that is observed just beyond the second reduction event in the CV of **1-Cu**. Indeed, CV with variable concentrations of **1-Cu** and H_2_O suggests first-order behavior in both of these reagents, consistent with this hypothesis (Figure S20). Regardless of the exact reduced speciation of **1-Cu**, the spectroscopy and calculations support a vital role for ligand redox noninnocence as an electron storage/shuttling mechanism for catalysis. This echoes possible roles for the multi-Cu cluster in biological N_2_O reduction and may point to a more fundamental requirement for additional redox cofactors in N_2_O reduction catalysis by Cu.

## Conclusion

We report the first example of a molecular Cu catalyst for N_2_O reduction. This complex, **1-Cu**, enables electrocatalytic reduction of N_2_O with water to afford N_2_ with a high Faradaic efficiency. Catalytic reduction with a chemical reducing agent was also demonstrated with dilute Na/Hg. Electrochemical studies support the onset of catalysis after reduction of **1-Cu** twice, and a combination of spectroscopy and theory supports the importance of ligand-based reductions in forming reduced intermediates. While **1-Cu** is a highly unusual example of a Cu-based catalyst for N_2_O reduction, we do note that previous molecular electrocatalysts with other transition metals show higher Faradaic efficiencies (>90%) and less decomposition.^43−48^ For instance, [Re(2,2′-bipyridine)(CO)_3_Cl] boasts ∼200 turnovers over the course of 2 h with no significant catalyst degradation, albeit with a comparatively precious metal.^48^ The findings reported here provide an initial proof-of-concept validation for further efforts toward the design of new Cu-based molecular electrocatalysts for N_2_O reduction. In addition to improved performance metrics and increasing the stability of the active catalyst, there remain interesting mechanistic questions surrounding both electron transfer principles and details about N_2_O binding, reduction, and protonation events.

## Supporting Information Available

Experimental procedures, NMR spectra, electrochemistry data, TCD GC spectra and calibration curves, UV–visible spectra, EPR spectra, SXRD data, and DFT calculations (PDF)
**1-Cu** (CIF)
**1-Zn** (CIF)
XYZ files of three-coordinate Cu(I) with and without a reduced ligand, four-coordinate Cu(I) with and without a reduced ligand, Cu(II) with and without OTf, and OTf (ZIP)

## Supplementary Material

cs3c02658_si_001.pdf

cs3c02658_si_002.cif

cs3c02658_si_003.cif

## Accession Codes

CCDC 2266810 and 2266811 contain the supplementary crystallographic data for this paper. These data can be obtained free of charge via www.ccdc.cam.ac.uk/data_request/cif, or by emailing data_request@ccdc.cam.ac.uk, or by contacting The Cambridge Crystallographic Data Centre, 12 Union Road, Cambridge CB2 1EZ, UK; fax: + 44 1223 336033.
