# Supplementary material for: Electrochemical Reduction of N2O with a Molecular Copper Catalyst
Source: ACS Catal. 2023 Sep 14;13(19):12673–80. doi: 10.1021/acscatal.3c02658 (PMC10563017; doi:10.1021/acscatal.3c02658)
Supplement: Supplementary file 1 — cs3c02658_si_001.pdf [file cs3c02658_si_001.pdf]

Supplementary Materials for

Electrochemical Reduction of N_2O with a Molecular Copper Catalyst

Jorge L. Martinez[a], Joseph E. Schneider[a], Sophie W. Anferov[a], John S. Anderson[a]*

[a]Department of Chemistry, University of Chicago, Chicago, Illinois 60637, United States.

*Correspondence to: jsanderson@uchicago.edu

Table of Contents

Materials and Methods 3
General Considerations 3
Electrochemical Measurements 3
Gas Chromatography (GC) 4
Calculating Faradaic Efficiency (FE) 4
Synthesis of Compounds 4
Chemical Reductions with Na/Hg 6
NMR Spectroscopy 7
Cyclic Voltammetry 14
Controlled Potential Electrolysis (CPE) 21
NMR Analysis After Controlled Potential Electrolysis (CPE) 23
TCD GC 25
Controlled Potential Electrolysis (CPE) in the Absence of N_2O 27
UV-Vis Spectroscopy 29
EPR spectroscopy 30
Single Crystal X-ray Diffraction (SXRD) 32
Density Functional Theory (DFT) 36

Materials and Methods

General Considerations

All manipulations were performed under a dry nitrogen atmosphere using either standard Schlenk techniques or in an M. Braun Unilab Pro glovebox unless otherwise stated. Glassware was dried at 180 °C for a minimum of two hours and cooled under vacuum prior to use. All chemicals were obtained from commercial sources and used as received unless otherwise stated. Solvents were dried on a solvent purification system from Pure Process Technologies before storing over 4 Å molecular sieves under N_2. Diethyl ether (Et_2O) was stirred over NaK alloy and passed through a column of activated alumina prior to storing over 4 Å sieves under N_2. Ultrapure water was obtained from a Milli-Q system. 2,6-$(Me_3Si)_2$Py was prepared according to a previously reported procedure.[1] EPR spectra were recorded on a Bruker Elexsys E500 spectrometer with an Oxford ESR 900 X-band cryostat and a Bruker Cold-Edge Stinger and data was analyzed using the EasySpin Matlab suite.[2] UV–vis spectra were recorded on a Thermo Scientific Evolution 300 spectrometer with the VISIONpro software suite. A standard 1 cm quartz cuvette with an airtight screw cap with a puncturable Teflon seal was used for all measurements. NMR spectra were recorded on either Bruker DRX-400 or AVANCE-500 spectrometers. Single crystal X-ray diffraction data was collected at the Advanced Photon Source of Argonne National Laboratory (beamline 15- ID B,C,D) using X-ray radiation with a wavelength of λ=0.41328 Å. Combustion analysis was performed by Midwest Microlab.

Electrochemical Measurements

Electrochemical measurements were carried out using a BAS Epsilon potentiostat and using BAS Epsilon software version 1.40.67NT. Cyclic voltammetry measurements were collected in an undivided three-electrode setup using a 3-mm diameter glassy carbon working electrode, a Pt wire counter electrode, and a Ag wire pseudo-reference electrode. Potentials were referenced by adding ferrocene (1 mM) as an internal standard.

Controlled potential electrolysis (CPE) experiments were performed in a custom-made airtight two-chamber H-cell with a pressure equalizing arm separated by a fine glass frit. A conventional three-electrode set-up for CPE was carried out using RVC as the working electrode, a Ag^+/Ag (10 mM $AgBF_4$ + 0.1 M $N^nBu_4PF_6$ in MeCN) reference electrode and a sacrificial Zn rod as the counter electrode separated by a glass frit containing 0.1 M $N^nBu_4PF_6$ in MeCN. The working electrode (Duocel RVC Foam Panel, 100 PPI, 3% relative density) was assembled by piercing the RVC foam (cut to approximately 0.5 x 0.5 x 2 mm^3) with a carbon rod. A conductive carbon glue was used as an adhesive between the carbon rod and RVC and the electrode was dried in an oven overnight. Approximately half of the RVC electrode submerged into solution avoid contact the carbon rod during CPE. The solutions in both chambers were sparged with N_2O for 1 h and the headspace was purged for an additional 30 min prior to electrolysis to minimize the amount of trace N_2 in the system. A flask containing dry acetonitrile was used as a pre-bubbler during sparging to reduce solvent loss. The cell was covered in aluminum foil and stirred at 720 rpm during electrolysis.

Gas Chromatography (GC)
Headspace gas sampling was analyzed using an Agilent 7890B GC equipped with both a flame ionization detector (FID) and a thermal conductivity detector (TCD). TCD chromatograms were collected for the detection of N_2 using He as a carrier gas through a CARBOXEN™ 1010 PLOT fused silica capillary column (30m x 0.32 mm). A calibration curve for product quantification was made by sampling known N_2/N_2O mixtures. The H-cell was allowed to sit while stirring (720 rpm) for 1 h after sparging and after electrolysis to guarantee gas homogeneity in the headspace.

Injection volume 100 μL; He carrier gas, flow 1 mL/min; Inlet 150 °C; Oven: 35 °C (15 min) then 24 °C /min to 220 °C (hold 8 min); Split ratio 30:1; TCD detector 230 °C.

Calculating Faradaic Efficiency (FE)
Faradaic efficiency (FE) was determined similarly to a previous report where FE was calculated based on the amount of N_2 found in the headspace since the amount of N_2 in solution was determined to be negligible (6.4 μM in acetonitrile).[3] The volume of N_2 in the headspace after 1 h of controlled potential electrolysis was determined as follows:

The area of a GC measurement from a 100 μL sample of the headspace before electrolysis (A_{N2}^{sys}) was subtracted from the area after electrolysis (A_{N2}^{elec}), $A_{N2}^{tot} = A_{N2}^{sys} - A_{N2}^{elec}$. Using a calibration curve, the amount of N_2 in the 100 μL sample was determined and thus the % of N_2 from measured can be obtained ($\%N_2 = \frac{\mu L\ N2}{100\ \mu L} \times 100\%$). The total volume of N_2 in the headspace is obtained by:

$$V_{N2}(mL) = (8.8\, mL \times \%N_2) \div 100$$

Where 8.8 mL is the total headspace volume. The Faradaic efficiency for N_2 is hence calculated using the equation:

$$FE\ (\%) = \left(\frac{2\ \times\ m_{N2}\ \times\ F}{Q}\right) \times 100\ \%$$

Where m_{N2} is the moles of N_2 produced determined by $m_{N2} = V_{N2}(mL) \div (22400\ mL\ mol^{-1})$, Q (in C) is the amount of charge passed, and F is Faraday's constant ($96500\ C\ mol^{-1}$).

Synthesis of Compounds

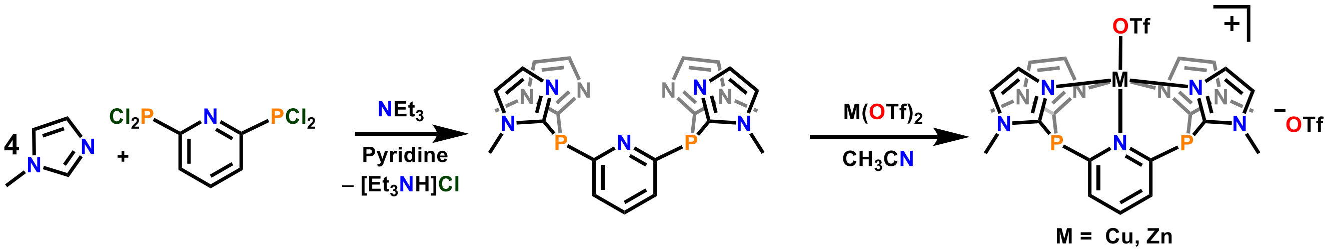

Preparation of 2,6-(Cl₂P)₂Py. Neat 2,6-(Me_3Si)$_2$Py (8.50 g, 34.3 mmol) was slowly added to a 500 mL Schlenk flask containing 30 mL of PCl_3. The reaction vessel was heated under reflux (80 °C) for one week. Volatiles were removed under reduced pressure and the crude mixture was

extracted in Et_2O. The solution was filtered through Celite and the solvent was removed under reduced pressure to afford an orange-yellow oil that was used in the next step without further purification (9.10 g). Samples of higher purity can be obtained by distillation under vacuum (T = 160 °C), affording a pale-yellow oil in 70% yield.

^{1}H NMR (400 MHz, CD_2Cl_2, 25 °C) δ = 8.25 (d, $^1J_{HH}$ = 7.83 Hz, 2H, Py-3,5-*H*), 8.12 (tt, $^1J_{HH}$ = 7.84 Hz, $^2J_{HP}$ = 1.57 Hz, 1H, Py-4-*H*).

^{13}C{^{1}H} NMR (400 MHz, CD_2Cl_2, 25 °C) δ = 163.97, 139.00, 126.88.

^{31}P{^{1}H} NMR (400 MHz, CD_2Cl_2, 25 °C) δ = 136.3 (s, 2P, Py-2,6-*P*).

$MeIm_4P_2Py$ (1). Neat 2,6-$(Cl_2P)_2$Py (9.00 g, 32.0 mmol) was added at ambient temperature to a round bottom flask containing a stirring pyridine solution (50 mL) of *N*-methylimidazole (10.5 g, 128 mmol) and triethylamine (13.0 g, 128 mmol). A white solid precipitated and the reaction mixture was allowed to stir for 24 h. Volatiles were removed under reduced pressure and the solid was taken out of the glovebox and dissolved in chloroform (200 mL). The solution was washed with 200 mL of 1 M NaOH and the organic phase was collected. The aqueous phase was washed two more times with chloroform (200 mL) and the organic phases were combined and dried with Na_2SO_4. The solution was filtered and the solvent was removed under reduced pressure. The solid product was washed with 200 mL of THF to afford **1** as a white powder (9.20 g, 60%). Analytically pure samples were obtained by recrystallization from CH_2Cl_2-Et_2O. Anal. Calcd. for $C_{21}H_{23}N_9P_2{\bullet}(H_2O)_{0.5}$: C 53.39, H 5.12, N 26.68; Found: C 53.32, H 4.87, N 26.41.

^{1}H NMR (400 MHz, CD_2Cl_2, 25 °C) δ = 7.57 (tt, $^1J_{HH}$ = 7.81 Hz, $^2J_{HP}$ = 2.90 Hz, 1H, Py-4-*H*), 7.32 (dt, $^1J_{HH}$ = 7.79 Hz, $^2J_{HP}$ = 1.22 Hz, 2H, Py-3,5-*H*), 7.11 (s, 4H, Im-*H*), 7.03 (s, 4H, Im-*H*), 3.48 (s, 12H, Im-CH_3).

^{13}C{^{1}H} NMR (400 MHz, CD_2Cl_2, 25 °C) δ = 161.60, 142.35, 135.49, 130.55, 126.55, 125.42, 34.61.

^{31}P{^{1}H} NMR (400 MHz, CD_2Cl_2, 25 °C) δ = −45.0 (s, 2P, Py-2,6-*P*).

[($MeIm_4P_2Py$)Cu(OTf)][OTf] (1-Cu). An acetonitrile solution (5 mL) of $Cu(OTf)_2$ (234 mg, 0.647 mmol) was slowly added to a slurry of $MeIm_4P_2Py$ (300 mg, 0.647 mmol) in 5 mL of acetonitrile and stirred for 2 h at room temperature. The solution was filtered through Celite and volatiles were removed under reduced pressure to afford **1-Cu** as a light blue solid in quantitative yield. Crystals suitable for X-ray diffraction were grown at room temperature by layering Et_2O on a concentrated solution of **1** in acetonitrile (438 mg, 82%). Anal. Calcd. for $C_{23}H_{23}CuF_6N_9O_6P_2S_2$: C 33.48, H 2.81, N 15.28; Found: C 33.68, H 2.86, N 15.46.

^{1}H NMR (400 MHz, CD_3CN, 25 °C) δ = 10.06 (1H, Py-*H*), 8.55 (2H, Py-*H*), 2.61 (12H, Im-CH_3). The remaining Im-*H* protons were not observed.

^{19}F{^{1}H} NMR (400 MHz, CD_3CN, 25 °C) δ = −74 (6F, SO_3CF_3).

^{31}P{^{1}H} NMR (400 MHz, CD_3CN, 25 °C) δ = −48 (2P, Py-2,6-*P*).

[(MeIm$_4$P$_2$Py)Zn(OTf)][OTf] (1-Zn). An acetonitrile solution (5 mL) of Zn(OTf)$_2$ (118 mg, 0.324 mmol) was slowly added to a slurry of MeIm$_4$P$_2$Py (150 mg, 0.324 mmol) in 5 mL of acetonitrile and stirred for 4 h at 50 °C. The solution was allowed to cool to room temperature and was filtered through Celite. Volatiles were removed under reduced pressure to afford **1-Zn** as a white solid in quantitative yield. Crystals suitable for X-ray diffraction were grown at room temperature by layering Et$_2$O on a concentrated solution of **1-Zn** in acetonitrile (195 mg, 73%). Anal. Calcd. for $C_{23}H_{23}ZnF_6N_9O_6P_2S_2$: C 33.41, H 2.80, N 15.24; Found: C 33.63, H 2.93, N 15.40.

^{1}H NMR (400 MHz, CD$_3$CN, 25 °C) δ = 8.12 (m, 2H, Py-3,5-*H*), 7.97 (m, 1H, Py-4-*H*), 7.69 (d, $^1J_{HH}$ = 1.02 Hz, 4H, Im-*H*), 7.29 (dd, $^1J_{HH}$ = 4.20 Hz, $^2J_{HH}$ = 0.95 Hz, 4H, Im-*H*), 3.93 (s, 12H, Im-C*H*$_3$).

^{13}C{^{1}H} NMR (400 MHz, CD$_3$CN, 25 °C) δ = 156.24, 142.84, 139.60, 136.78, 130.83, 126.12, 35.63.

^{19}F{^{1}H} NMR (400 MHz, CD$_3$CN, 25 °C) δ = −79 (6F, SO$_3$C*F*$_3$).

^{31}P{^{1}H} NMR (400 MHz, CD$_3$CN, 25 °C) δ = −95 (2P, Py-2,6-*P*).

Chemical Reductions with Na/Hg

N$_2$O Reduction. An acetonitrile solution (10 mL) containing 1 mol % **1-Cu** (8.2 mg) and 18 μL of H$_2$O (100 mmol) was sparged with N$_2$O for 1.5 h inside a 25 mL Schlenk flask. A slight excess of 0.1 % Na/Hg (210 mmol) was prepared inside a separate 50 mL Schlenk flask under an N$_2$ atmosphere. The headspace was then evacuated for 15 min and the flask was purged with N$_2$O for 15 min. The acetonitrile solution was then cannula transferred using N$_2$O gas into the flask containing Na/Hg and the mixture was stirred overnight (16 h). A control experiment in the absence of **1-Cu** was also done following this same procedure. Quantification of N$_2$ was determined and measured using TCD GC; no other gaseous products were detected in the headspace.

EPR of reduced compounds. A solution of **1-Cu** (0.5 mM) and **1-Zn** (10 mM) were prepared in acetonitrile and added to approximately 100 equivalents of 20 % Na/Hg. Both reaction mixtures were stirred for 1 h to afford a red solution for the reduced Zn species and a brown solution for the doubly reduced Cu complex.

Although the nature of the decomposition product(s) were not extensively investigated, it is worth noting that there were observable changes in the solutions that indicated degradation of the samples. For example, in the Zn reduction reaction the red solution slowly turns colorless after 1 h. In the case of the Cu reduction reaction, precipitates begin to slowly form after 1 h.

NMR Spectroscopy

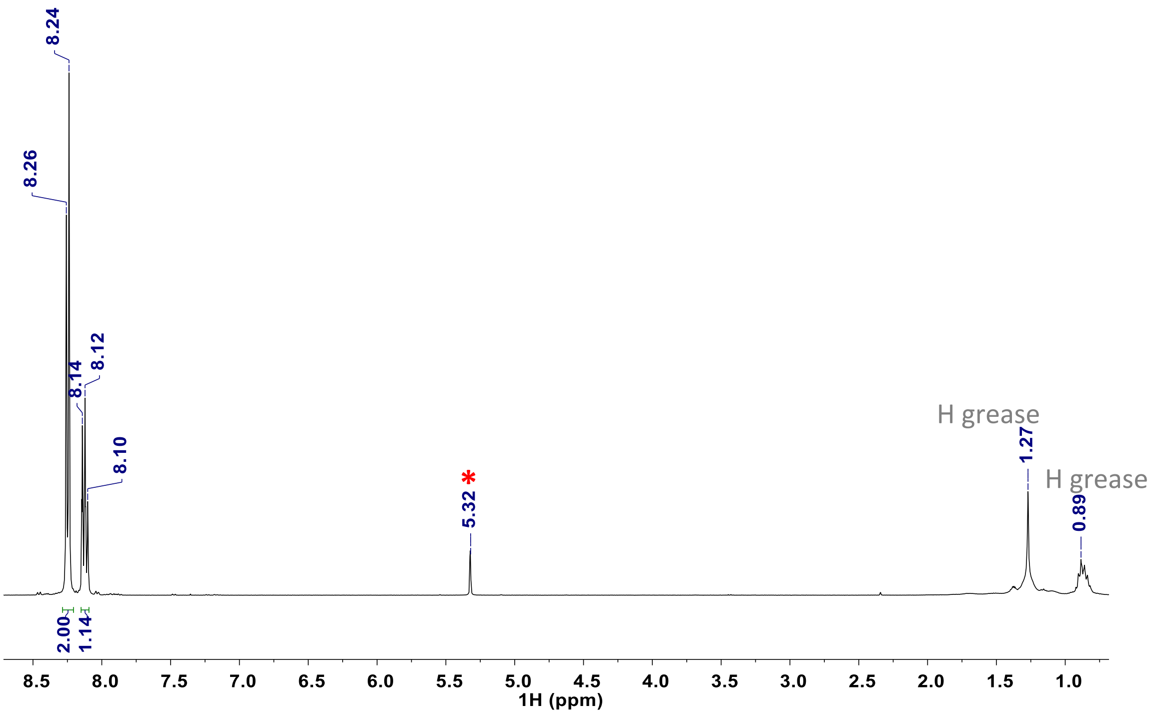

Figure S1. ^{1}H NMR spectrum of 2,6-($Cl_2P)_2Py$ in CD_2Cl_2 (*).

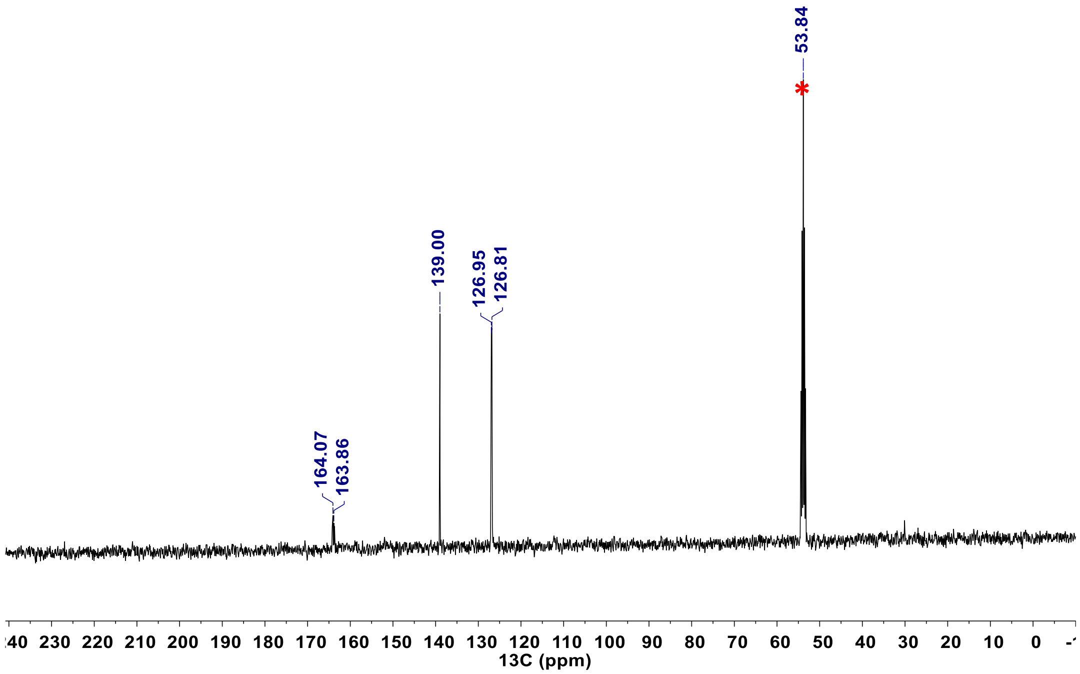

Figure S2. $^{13}C\{^{1}H\}$ NMR spectrum of 2,6-($Cl_2P)_2Py$ in CD_2Cl_2 (*).

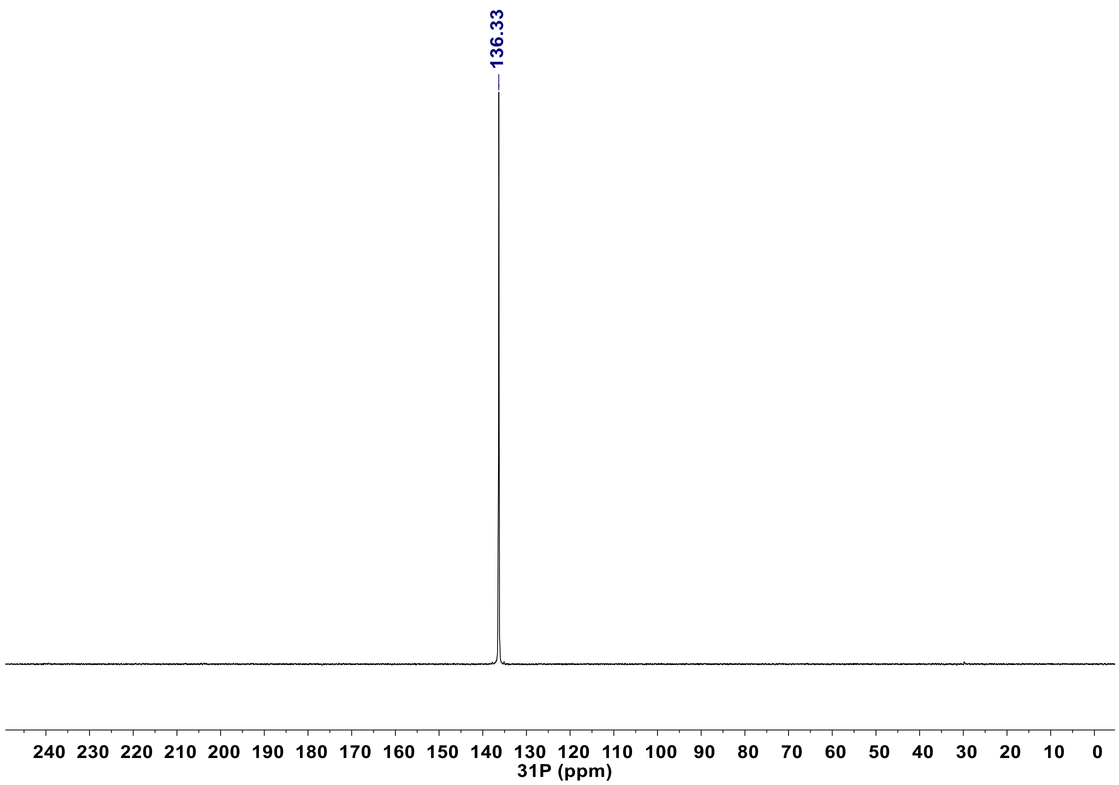

Figure S3. $^{31}P\{^1H\}$ NMR spectrum of 2,6-$(Cl_2P)_2Py$ in CD_2Cl_2.

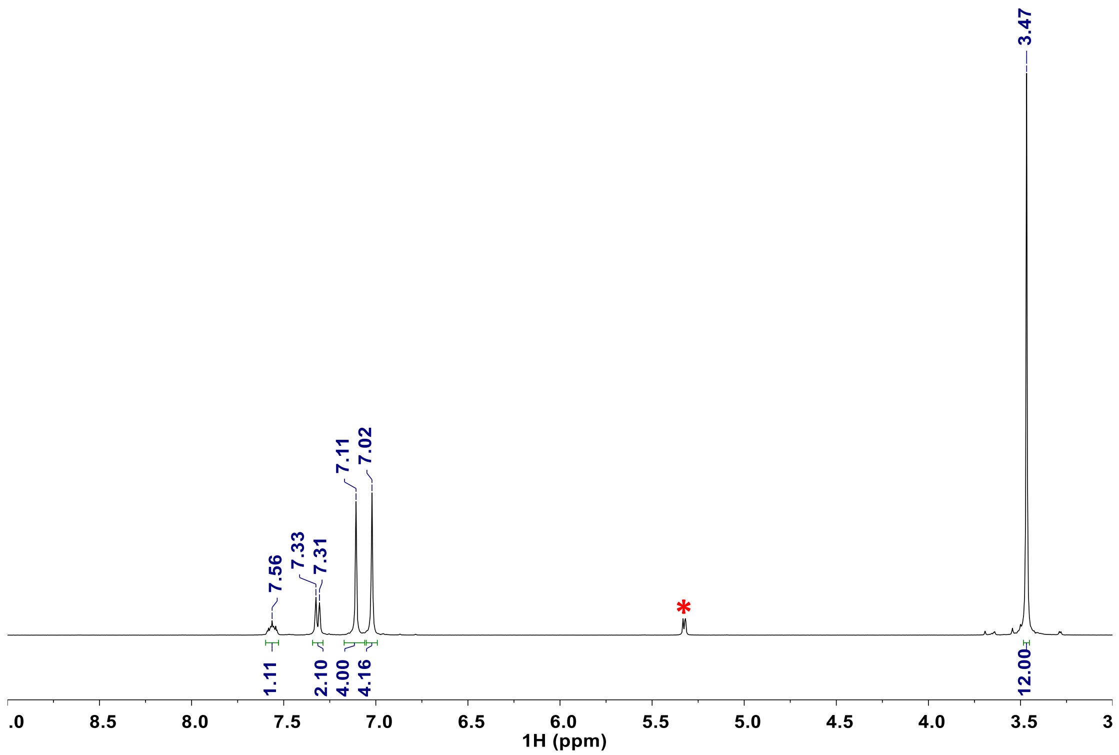

Figure S4. ^1H NMR spectrum of $MeIm_4P_2Py$ (**1**) in CD_2Cl_2 (*).

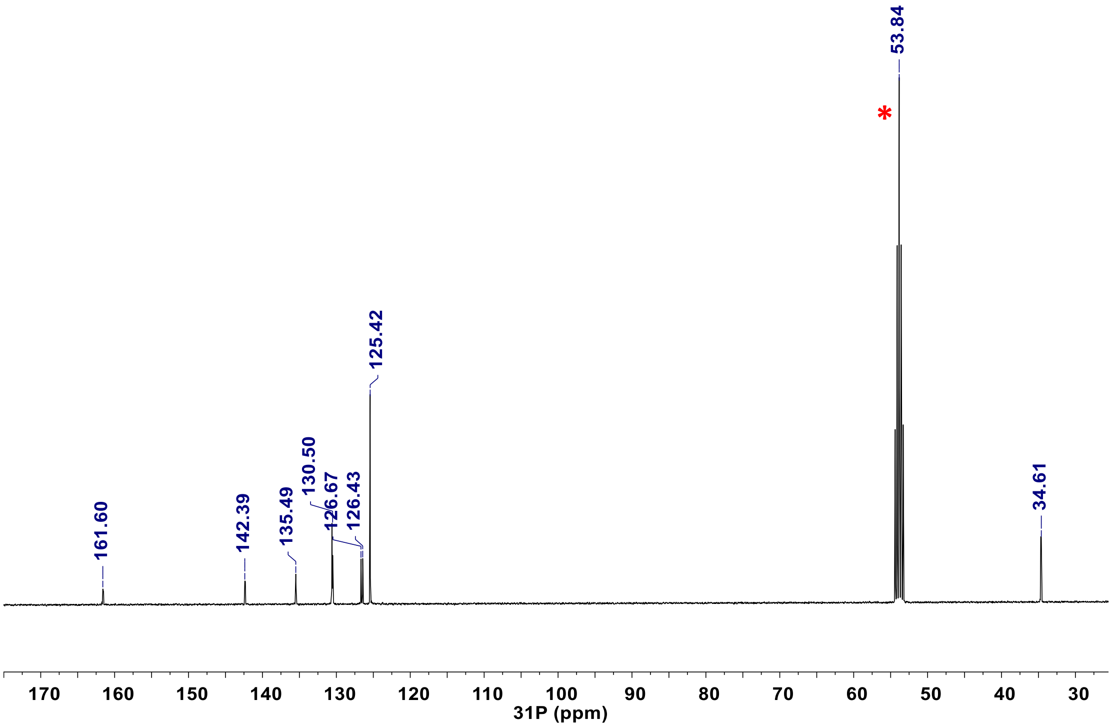

Figure S5. $^{13}C\{^{1}H\}$ NMR spectrum of $MeIm_4P_2Py$ (**1**) in CD_2Cl_2 (*).

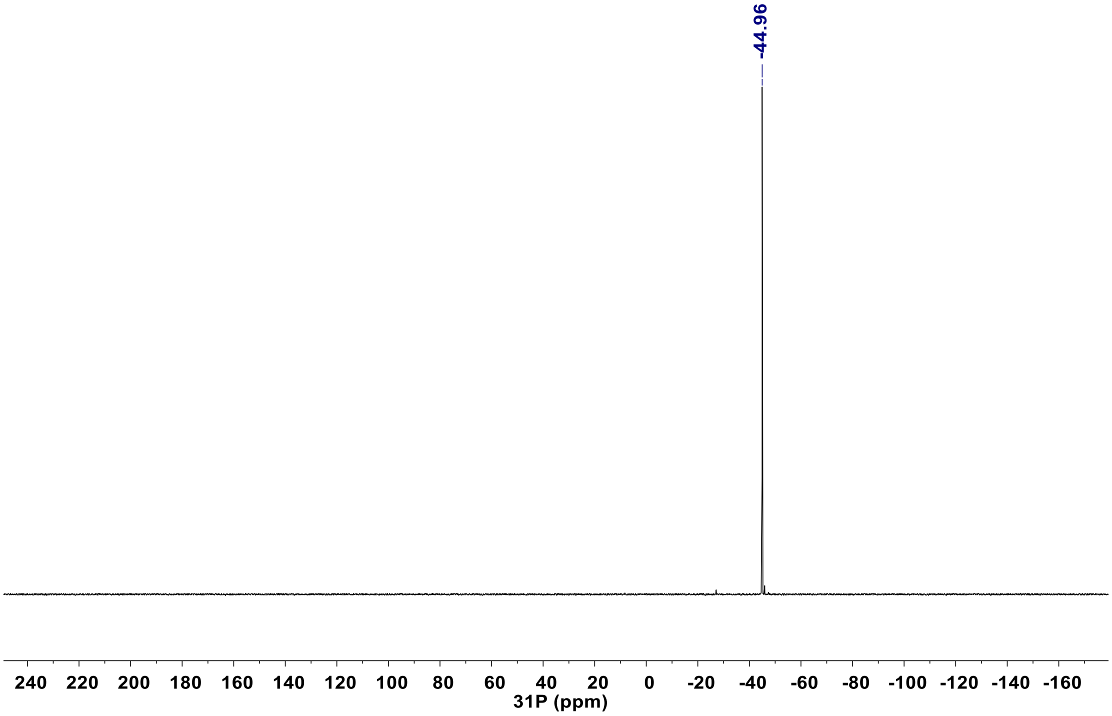

Figure S6. $^{31}P\{^{1}H\}$ NMR spectrum of $MeIm_4P_2Py$ (**1**) in CD_2Cl_2.

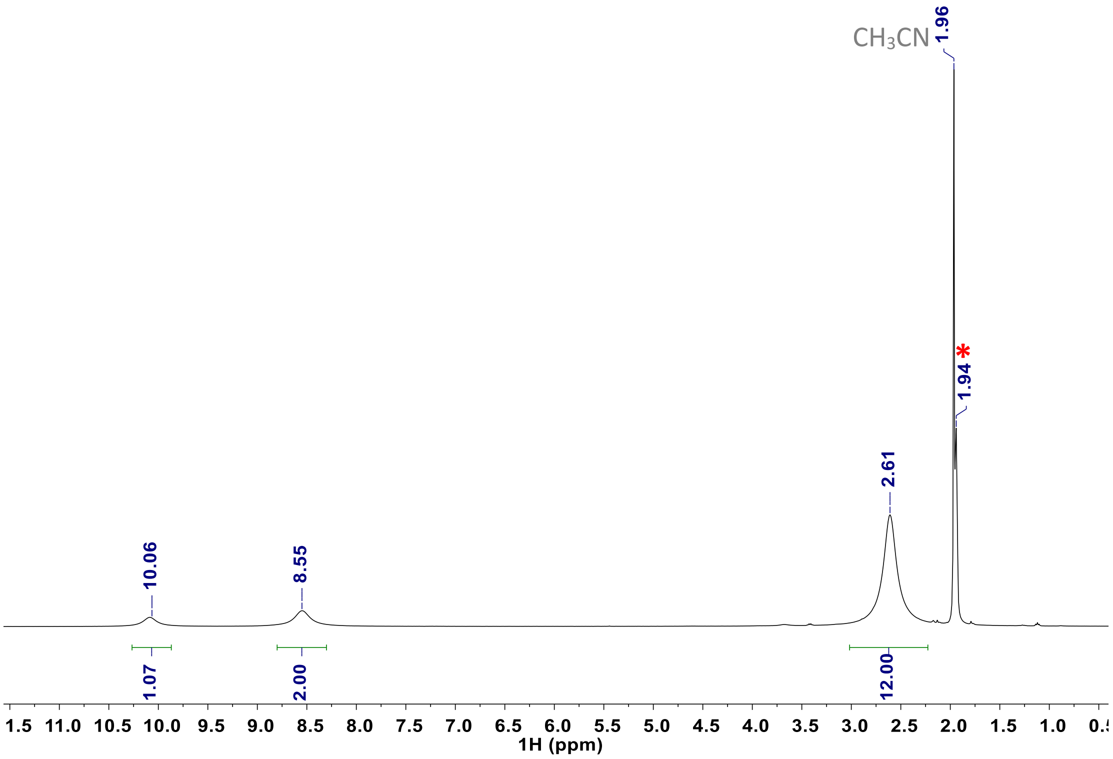

Figure S7. ^{1}H NMR spectrum of [(MeIm$_4$P$_2$Py)Cu(OTf)][OTf] (**1-Cu**) in CD_3CN (*). The remaining ImH protons were not observed.

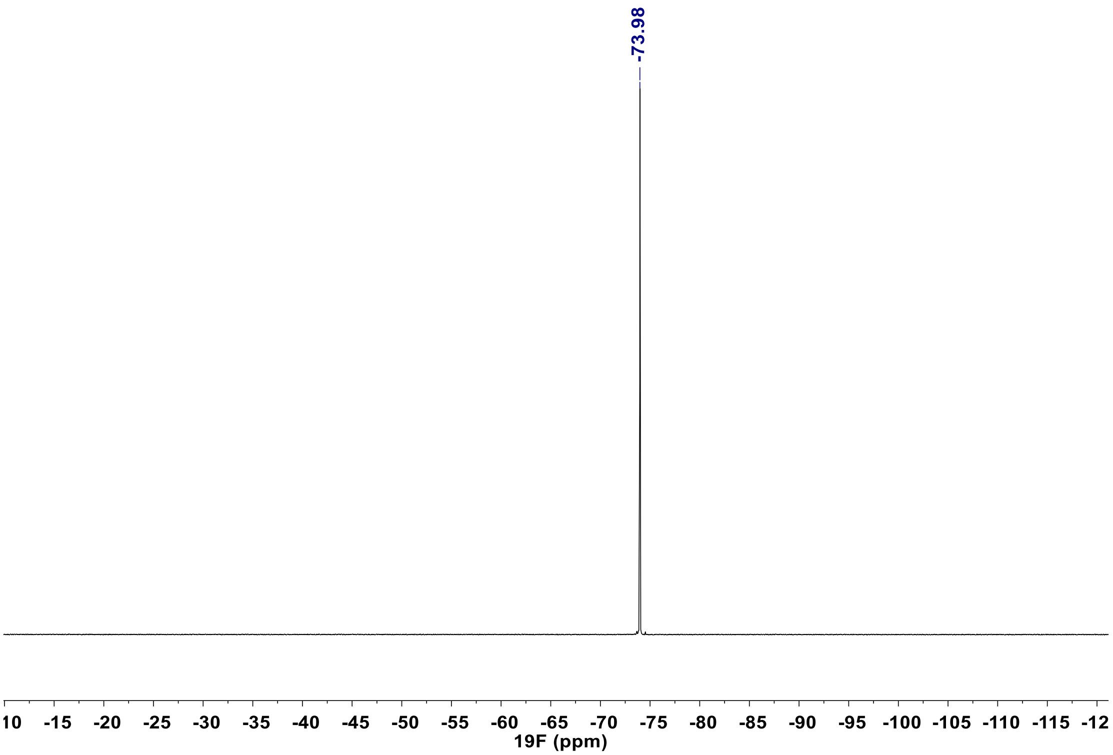

Figure S8. $^{19}F\{^{1}H\}$ NMR spectrum of [(MeIm$_4$P$_2$Py)Cu(OTf)][OTf] (**1-Cu**) in CD_3CN.

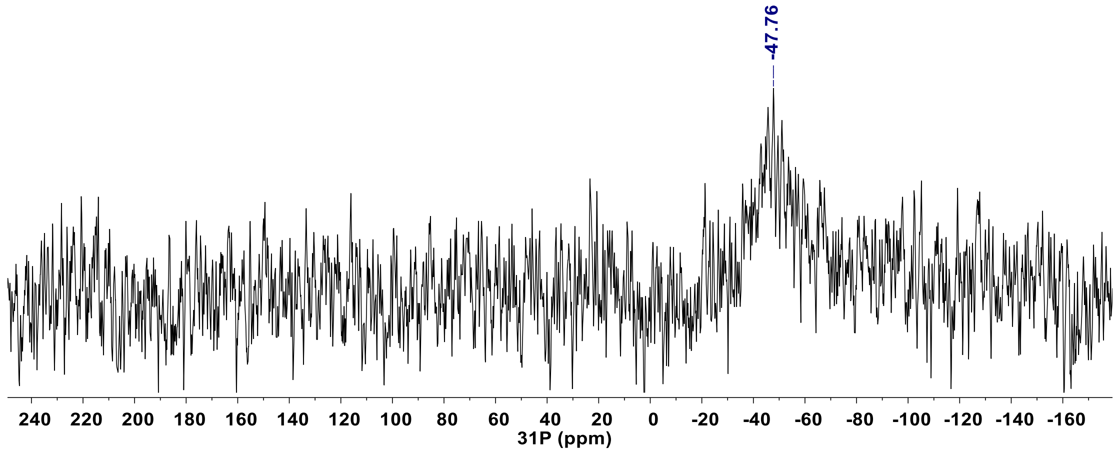

Figure S9. $^{31}P\{^{1}H\}$ NMR spectrum of [(MeIm$_4$P$_2$Py)Cu(OTf)][OTf] (**1-Cu**) in CD_3CN.

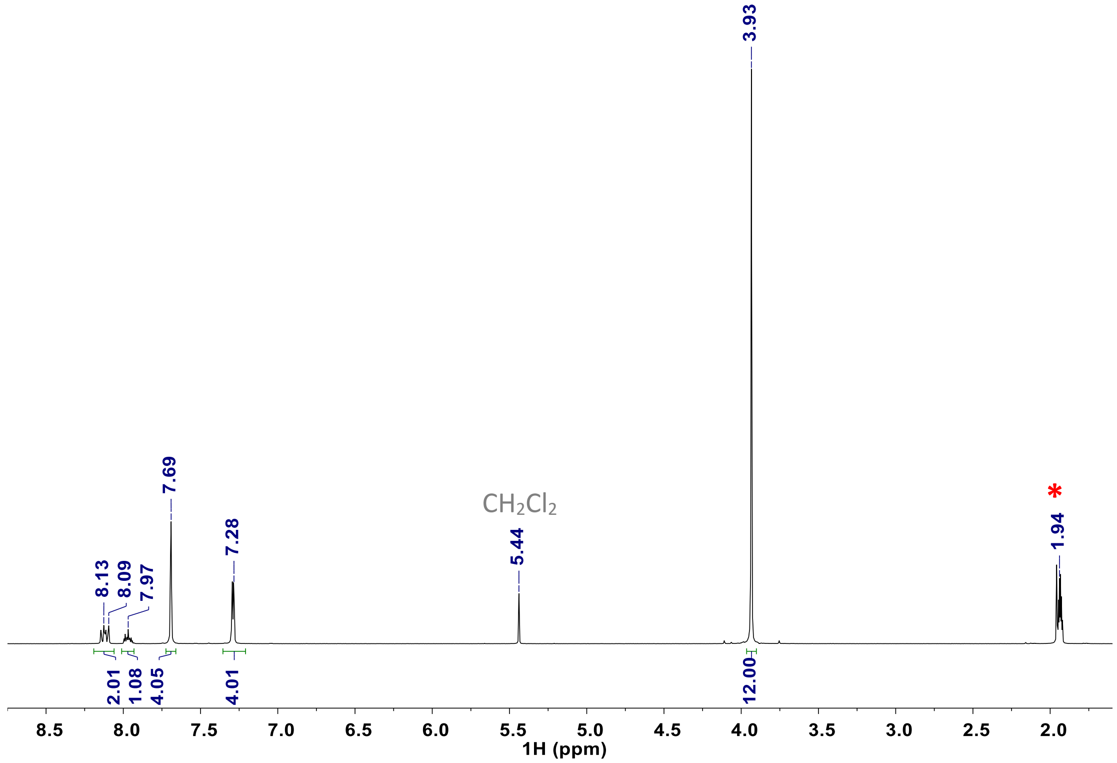

Figure S10. ^{1}H NMR spectrum of [(MeIm$_4$P$_2$Py)Zn(OTf)][OTf] (**1-Zn**) in CD_3CN (*).

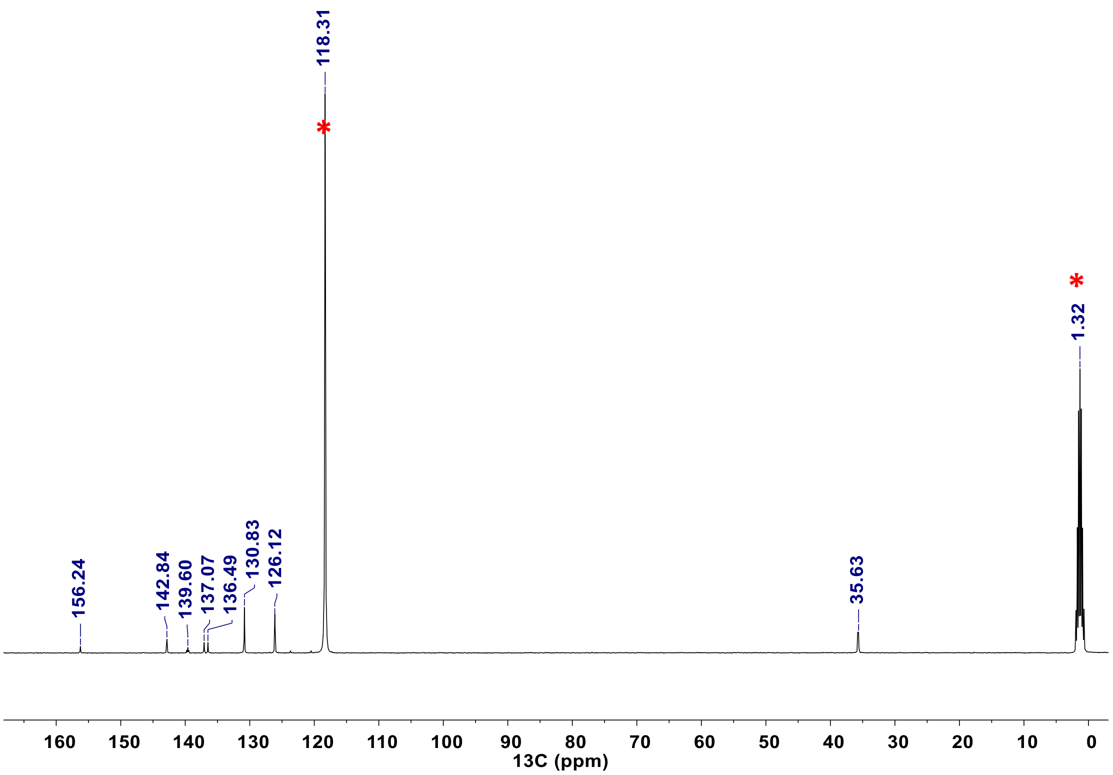

Figure S11. $^{13}C\{^{1}H\}$ NMR spectrum of [(MeIm$_4$P$_2$Py)Zn(OTf)][OTf] (**1-Zn**) in CD_3CN (*).

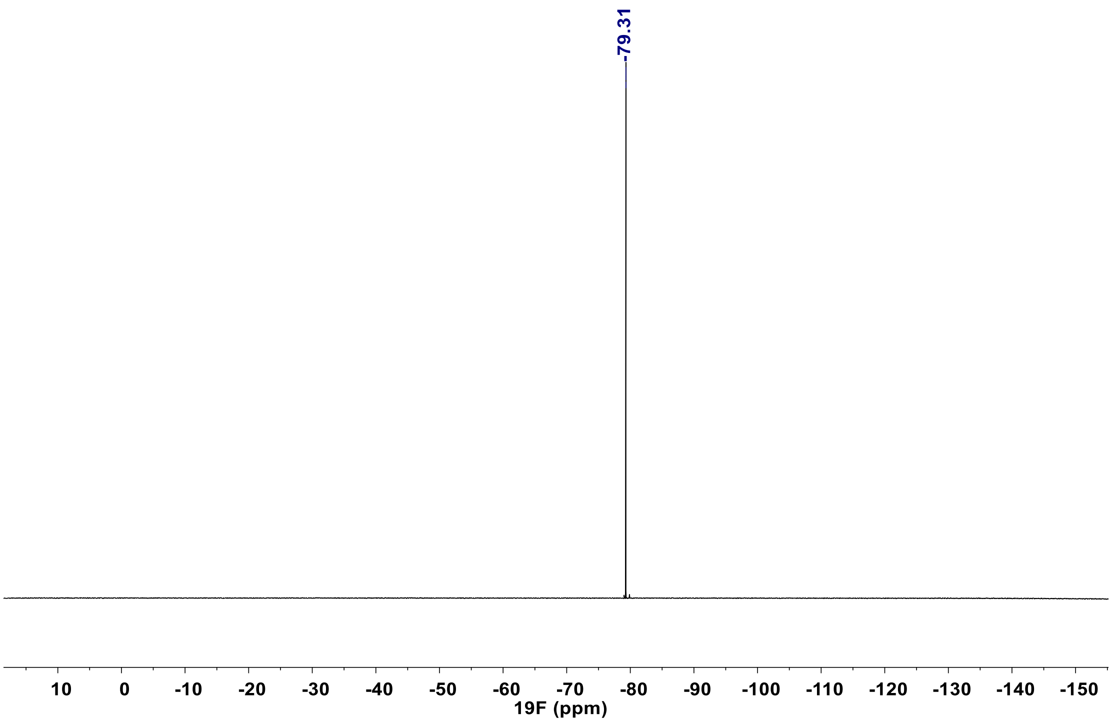

Figure S12. $^{19}F\{^{1}H\}$ NMR spectrum of [(MeIm$_4$P$_2$Py)Zn(OTf)][OTf] (**1-Zn**) in CD_3CN.

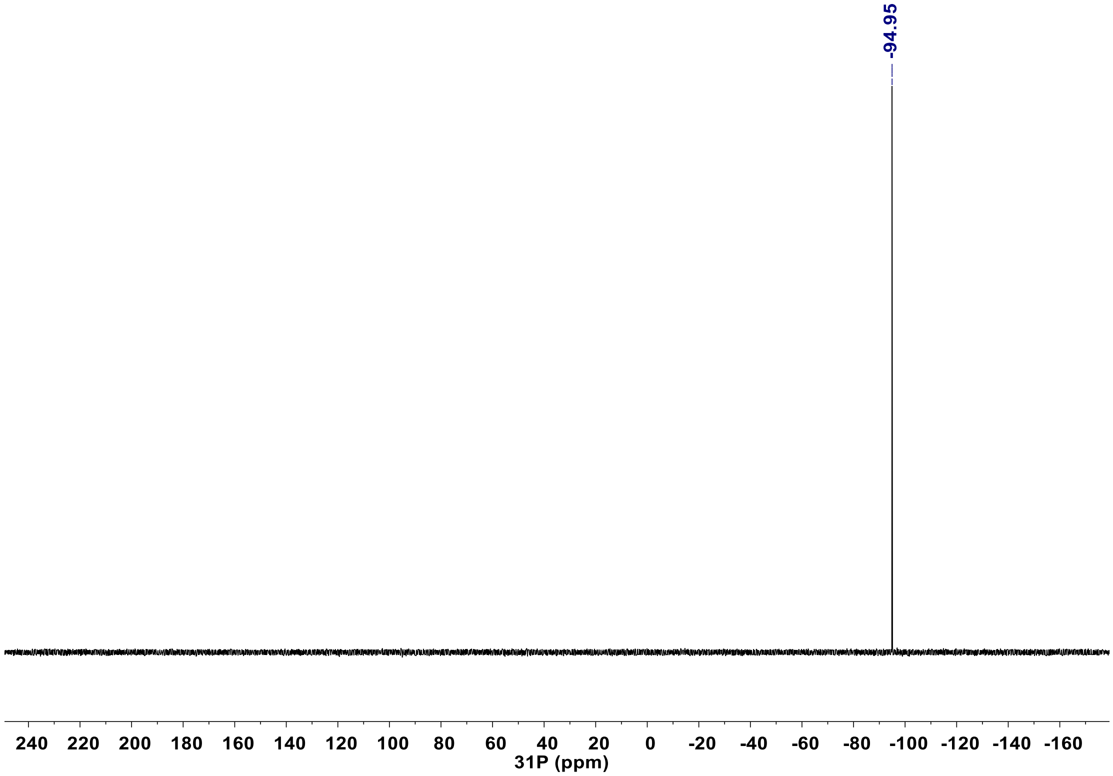

Figure S13. $^{31}P\{^{1}H\}$ NMR spectrum of [(MeIm$_4$P$_2$Py)Zn(OTf)][OTf] (**1-Zn**) in CD_3CN.

Cyclic Voltammetry

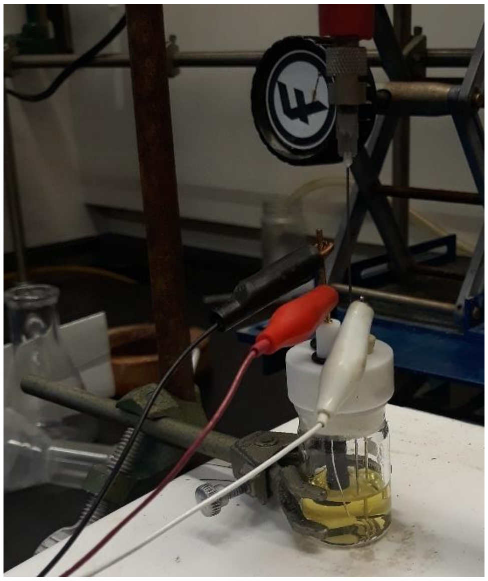

Figure S14. Typical setup for a three-electrode, one-compartment cell for cyclic voltammetry.

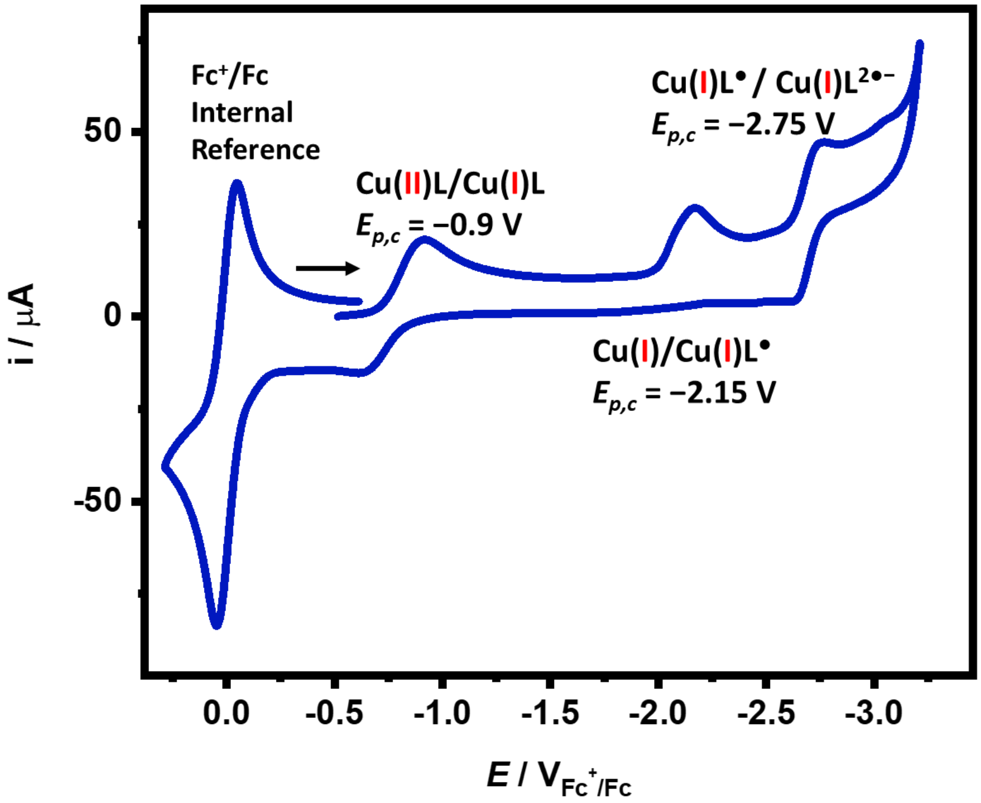

Figure S15. CV of **1-Cu** (1 mM) under N_2. Glassy carbon working electrode, Ag wire pseudo-reference electrode, Pt wire counter electrode; MeCN + 0.1 M $N^nBu_4PF_6$ supporting electrolyte, scan rate 100 mV s^{-1}.

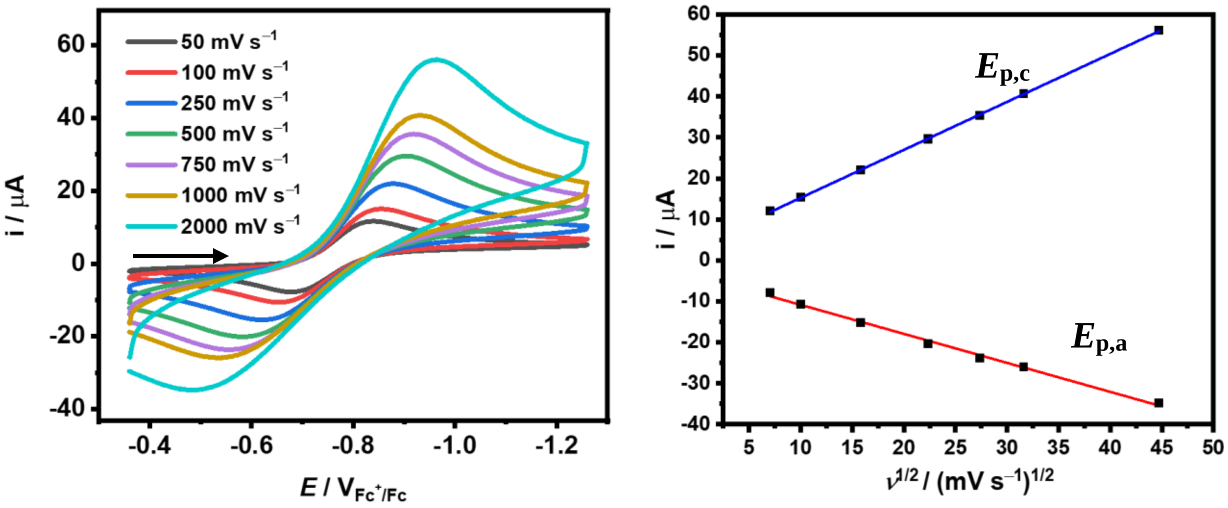

Figure S16. CV of **1-Cu** (1 mM) showing the scan rate dependence of the Cu(II)/Cu(I) redox couple. Glassy carbon working electrode, Ag wire pseudo-reference electrode, Pt wire counter electrode; MeCN + 0.1 M $N^nBu_4PF_6$ supporting electrolyte.

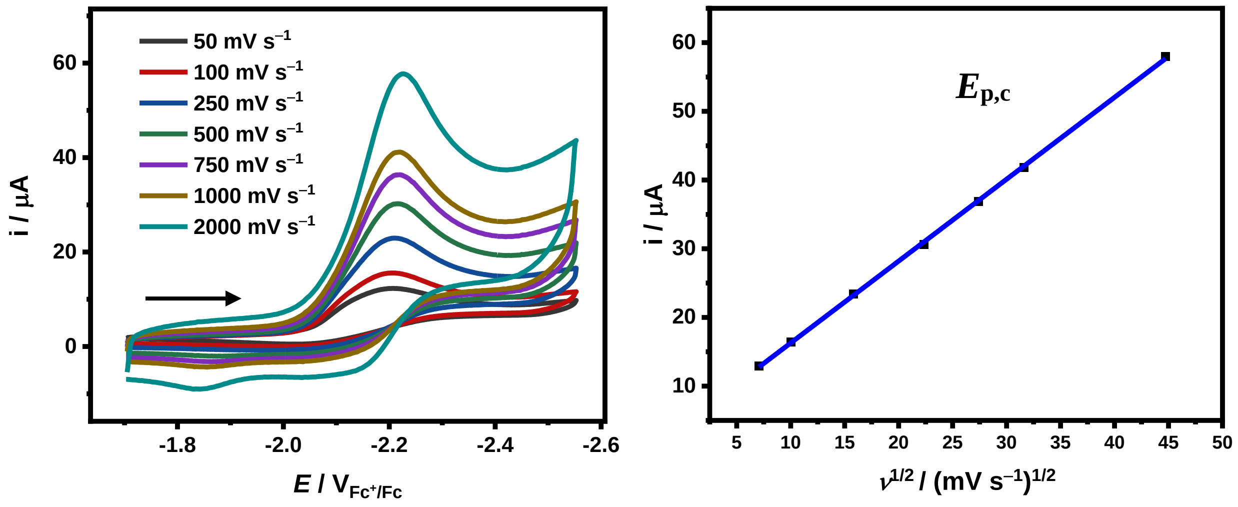

Figure S17. CV of **1-Cu** (1 mM) showing the scan rate dependence of the Cu(I)L^+/Cu(I)$L^\bullet$ redox couple (L = **1**). Glassy carbon working electrode, Ag wire pseudo-reference electrode, Pt wire counter electrode; MeCN + 0.1 M $N^nBu_4PF_6$ supporting electrolyte.

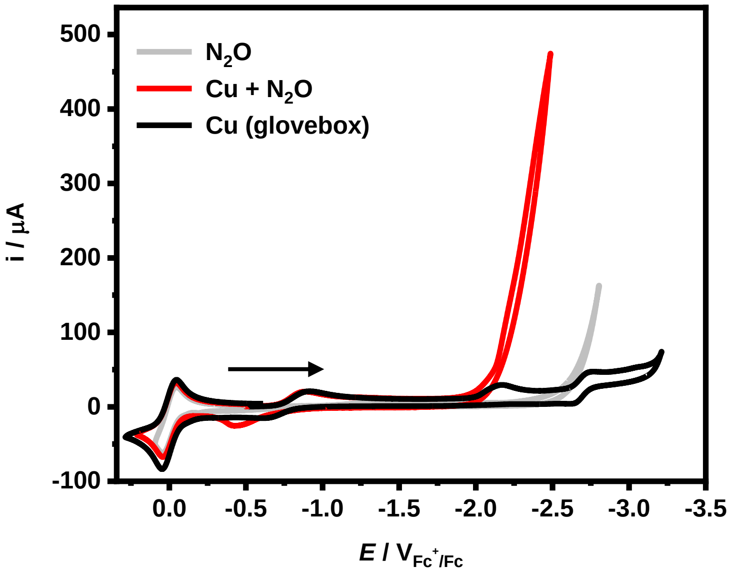

Figure S18. CV of **1-Cu** (1 mM) under N_2 (black), N_2O (red) and the direct reduction of N_2O with glassy carbon (gray). Glassy carbon working electrode, Ag wire pseudo-reference electrode, Pt wire counter electrode; MeCN + 0.1 M N^nBu_4OTf supporting electrolyte, scan rate 100 mV s^{-1}.

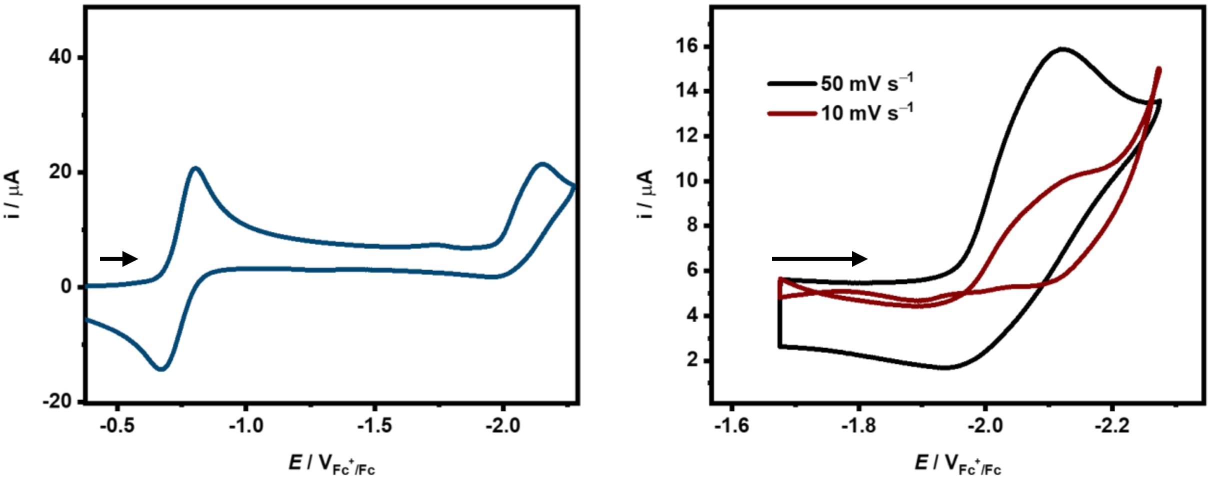

Figure S19. CV of **1-Cu** (1 mM) in a 95:5 MeCN-H_2O mixed solvent under N_2 (scan rate 100 mV s^{-1}). Working electrode was polished between scans. Glassy carbon working electrode, Ag wire pseudo-reference electrode, Pt wire counter electrode; MeCN + 0.1 M $N^nBu_4PF_6$ supporting electrolyte.

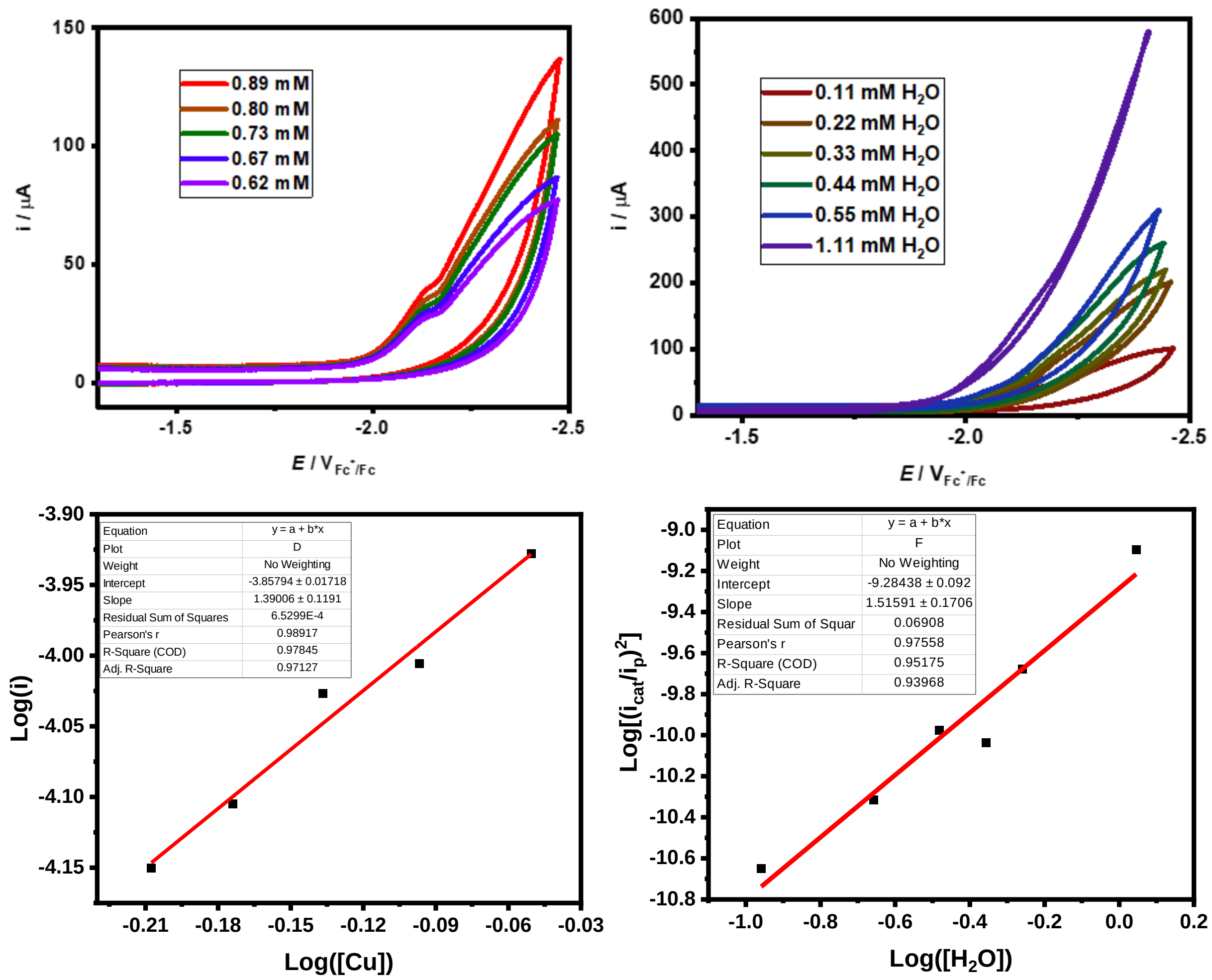

Figure S20. CVs of various concentrations under 1 atm of N_2O of (a) **1-Cu** (top) in the presence of 100 mM H_2O and (b) various concentrations of H_2O in the presence of 1 mM **1-Cu**. The logarithmic plots of the current at -2.4 V vs Fc^+/Fc with respect to (c) catalyst concentration and (d) H_2O concentration. The linear dependence in both plots suggests that the reaction is likely first order in catalyst and in H_2O. Glassy carbon working electrode, Ag wire pseudo-reference electrode, Pt wire counter electrode; MeCN + 0.1 M $N^nBu_4PF_6$ supporting electrolyte.

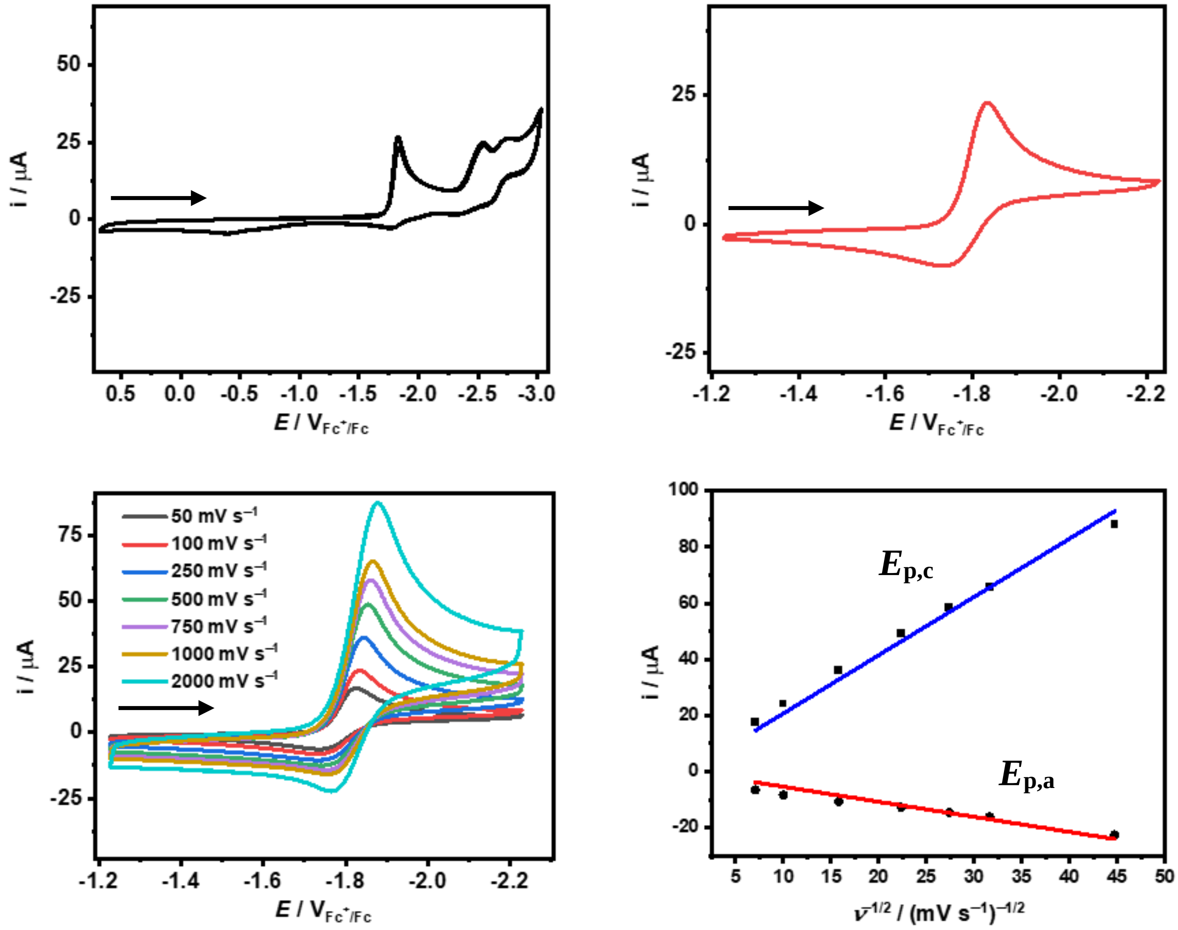

Figure S21. CV of **1-Zn** (1 mM). Glassy carbon working electrode, Ag wire pseudo-reference electrode, Pt wire counter electrode; MeCN + 0.1 M $N^nBu_4PF_6$ supporting electrolyte, scan rate 100 mV s^{-1}.

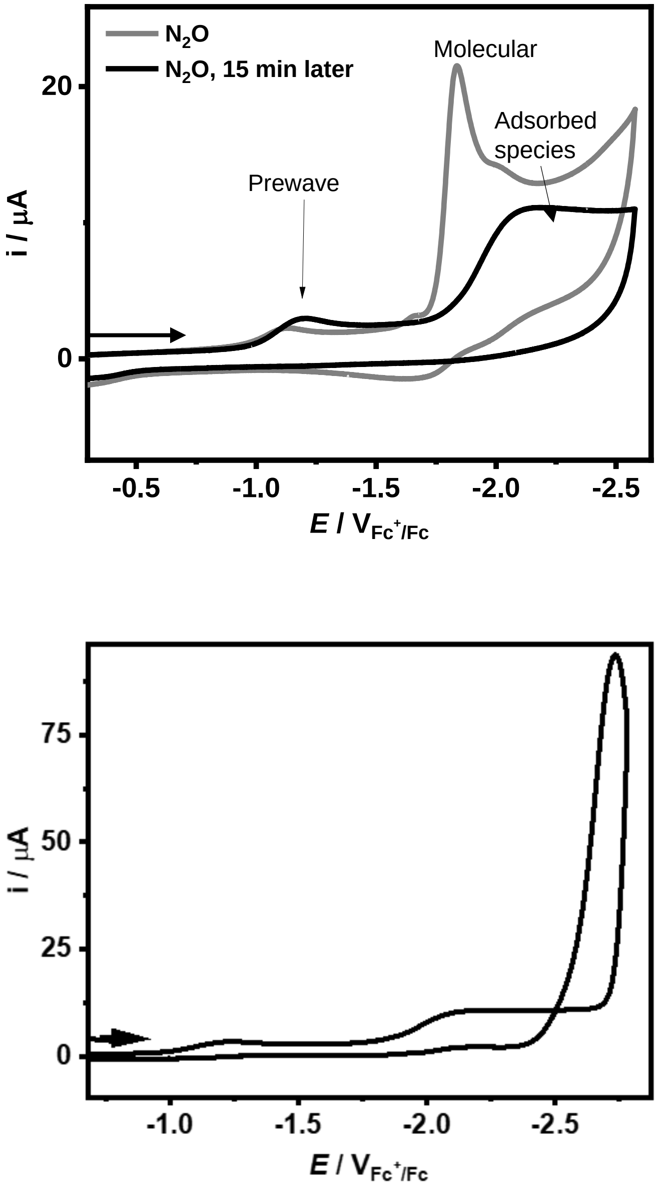

Figure S22. CV of **1-Zn** (1 mM) in the presence of H_2O (100 mM) under 1 atm N_2O. The redox couple associated with the molecular complex disappears over time with exposure to N_2O. The nature of the adsorbed species was not investigated. Glassy carbon working electrode was polished between scans. Glassy carbon working electrode, Ag wire pseudo-reference electrode, Pt wire counter electrode; MeCN + 0.1 M $N^nBu_4PF_6$ supporting electrolyte, scan rate 100 mV s^{-1}.

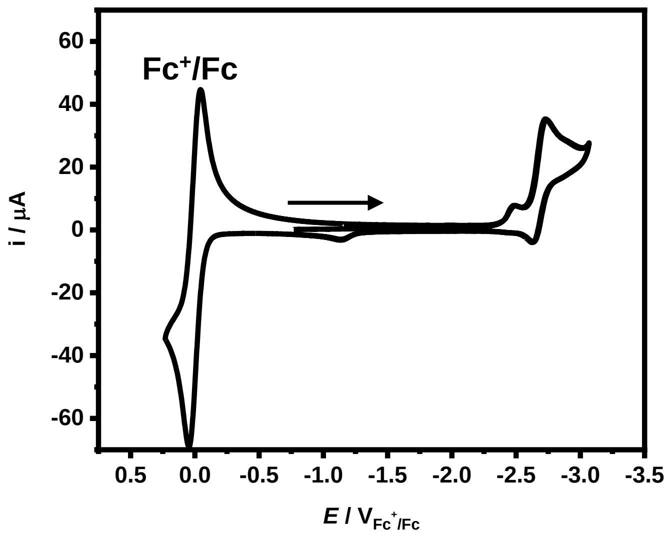

Figure S23. CV of **1** (1 mM) showing adsorption behavior as is suggested by the prewave at approximately −2.5 V. A ‘stripping’ wave which is likely associated with desorption of the species is observed when sweeping oxidatively at approximately −1.2 V. Glassy carbon working electrode, Ag wire pseudo-reference electrode, Pt wire counter electrode; MeCN + 0.1 M $N^nBu_4PF_6$ supporting electrolyte, scan rate 100 mV s^{-1}.

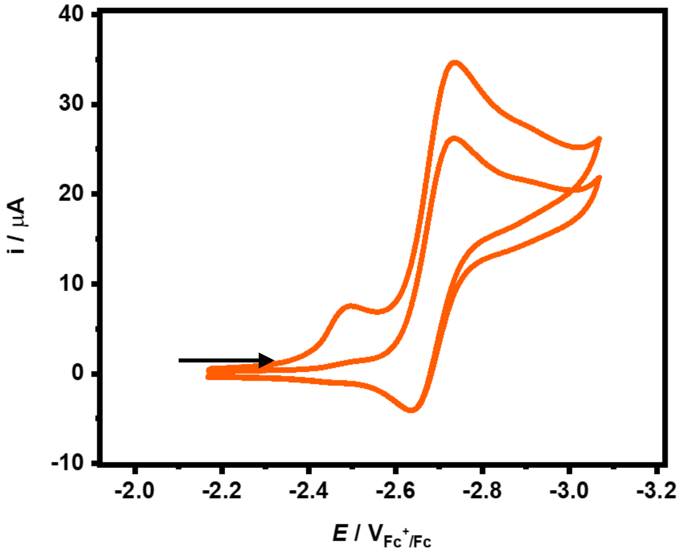

Figure S24. CV of **1** (1 mM) showing loss of the prewave feature upon second scan, consistent with adsorption. Glassy carbon working electrode, Ag wire pseudo-reference electrode, Pt wire counter electrode; MeCN + 0.1 M $N^nBu_4PF_6$ supporting electrolyte, scan rate 100 mV s^{-1}.

Controlled Potential Electrolysis (CPE)

(A)

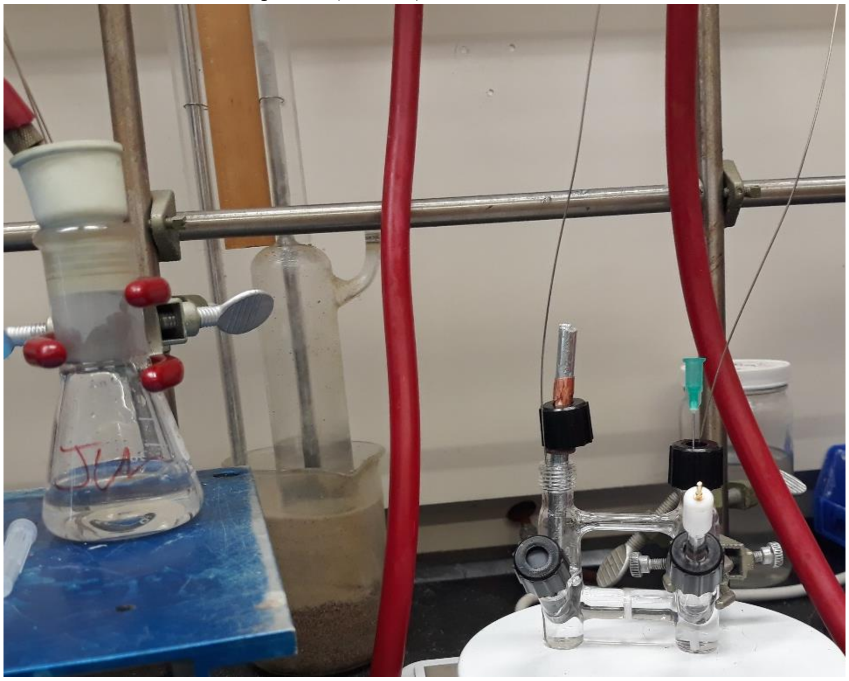

(B)

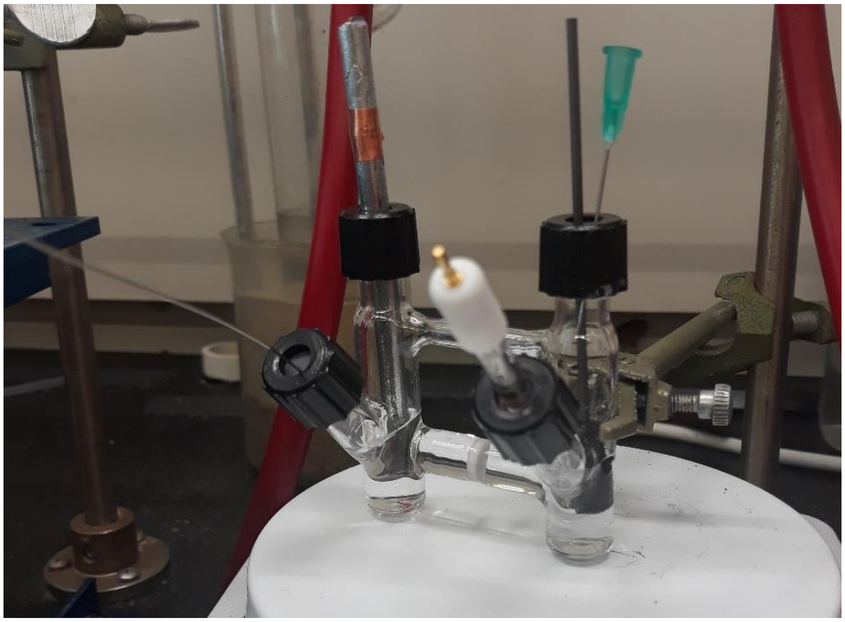

Figure S25. Two-compartment divided cell used for controlled potential electrolysis experiments. The compartment on the left contains a Zn rod counter electrode with conductive copper tape wrapped on top to clamp to the electrode alligator clips. The compartment on the right is the RVC working electrode and Ag^+/Ag reference electrode. (A) sparging of both working and counter compartment solutions with N_2O for 1h; (B) sparging headspace with N_2O for 30 min.

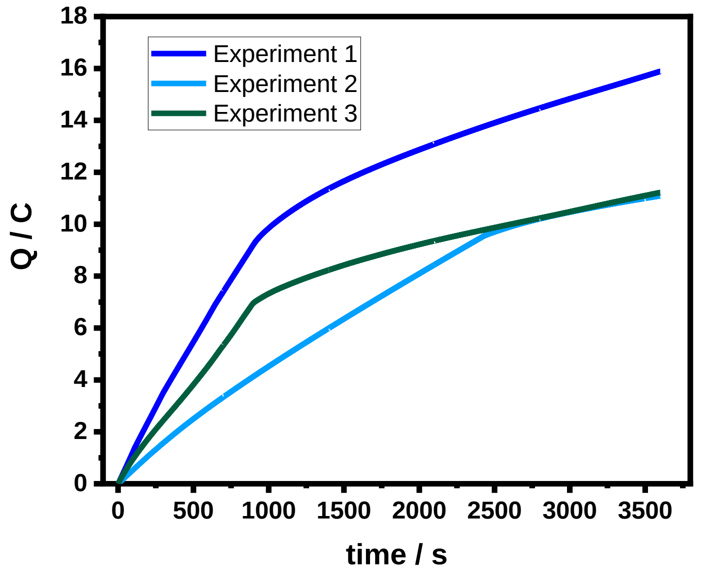

Figure S26. Q vs time of CPE experiments showing dependence on RVC working electrode surface area. RVC working electrode, Ag^{+}/Ag wire reference electrode, Zn rod counter electrode; MeCN + 0.1 M $N^{n}Bu_4PF_6$ supporting electrolyte.

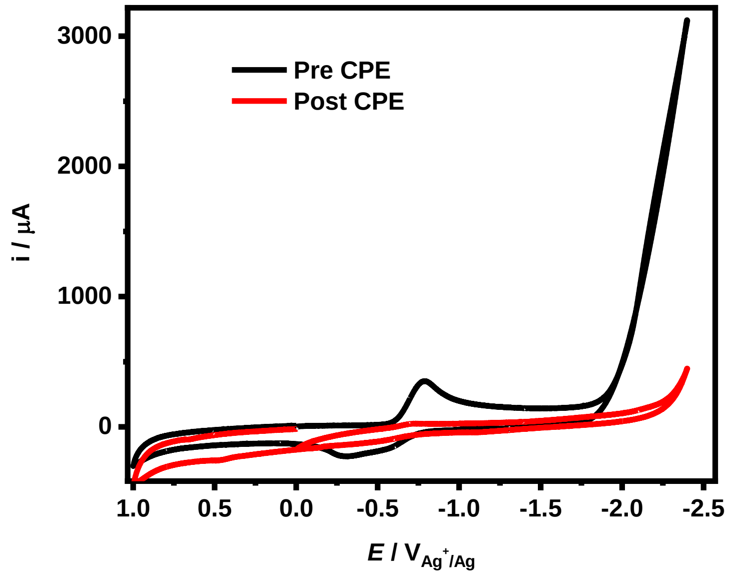

Figure S27. CV of **1-Cu** (1 mM) before (black trace) and after (red trace) controlled potential electrolysis (1 atm N_2O, 100 mM H_2O). No electrochemically active species or catalytic current is observed after CPE, suggesting that the decomposition product(s) are inactive towards N_2O reduction. Glassy carbon plate (1 cm^2) working electrode, Ag^{+}/Ag wire reference electrode, Zn rod counter electrode; MeCN + 0.1 M $N^{n}Bu_4PF_6$ supporting electrolyte, scan rate 100 mV s^{-1}.

NMR Analysis After Controlled Potential Electrolysis (CPE)

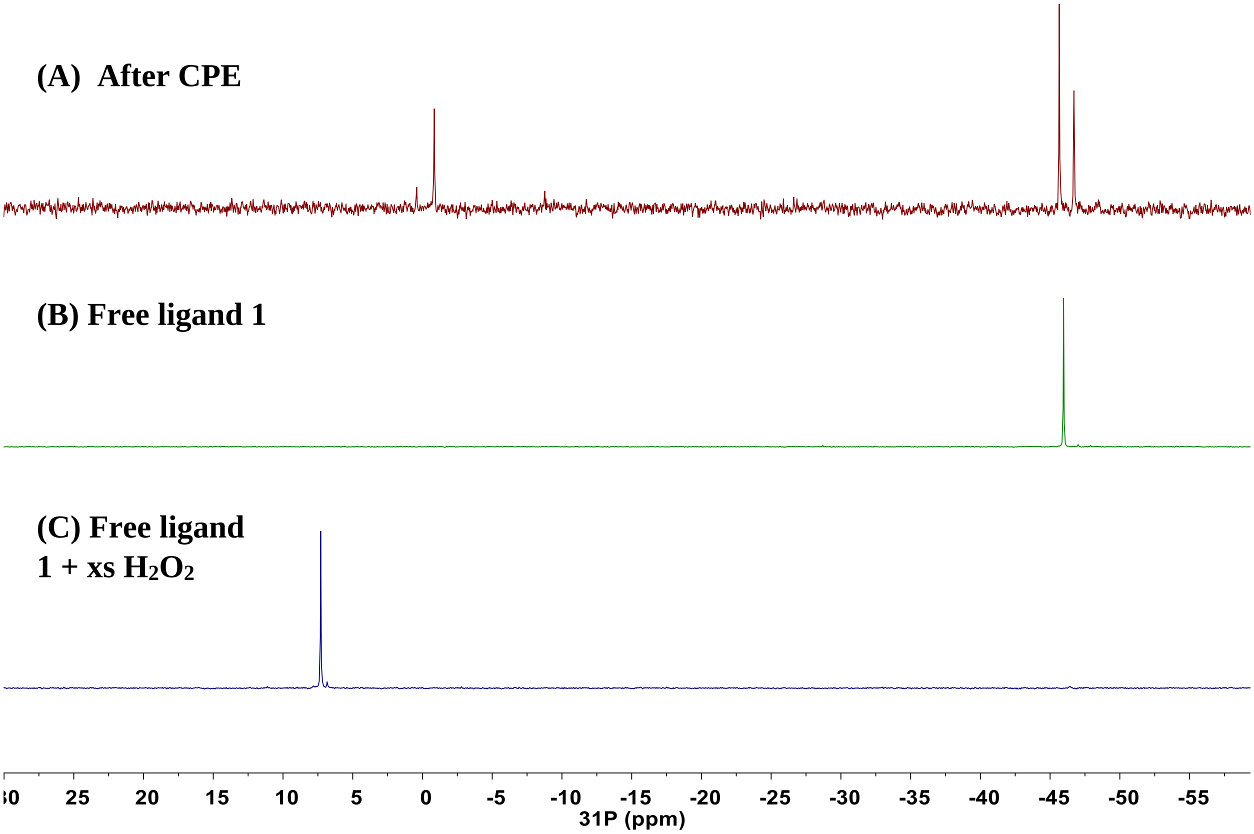

Figure S28. $^{31}P\{^1H\}$ NMR spectra (DMSO-d_6) of: (A) the solution after CPE; (B) Free ligand 1; (C) Free ligand in the presence of excess H_2O_2 (30% in H_2O). No evidence for phosphine oxide is observed post-electrolysis.

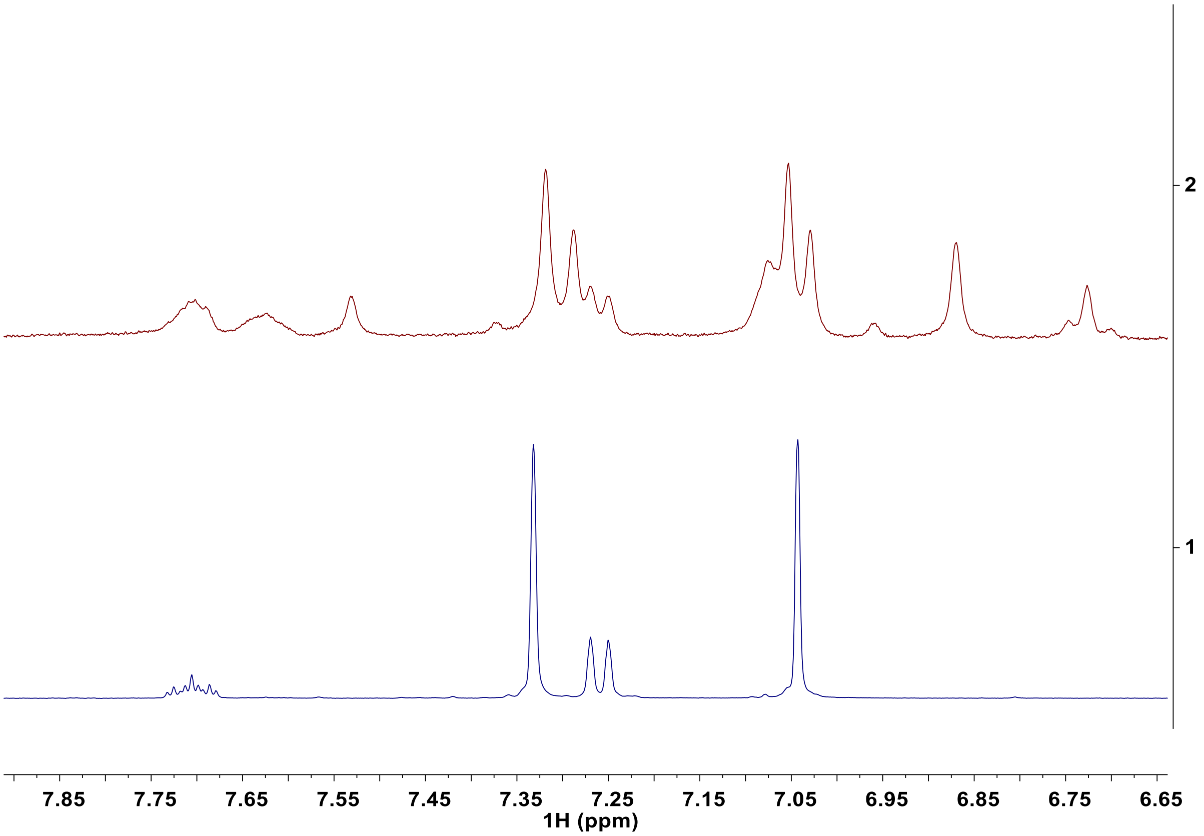

Figure S29. ^{1}H NMR spectra (DMSO-d$_6$) showing the aromatic region of the solution after CPE (red) and free ligand 1 (blue).

TCD GC

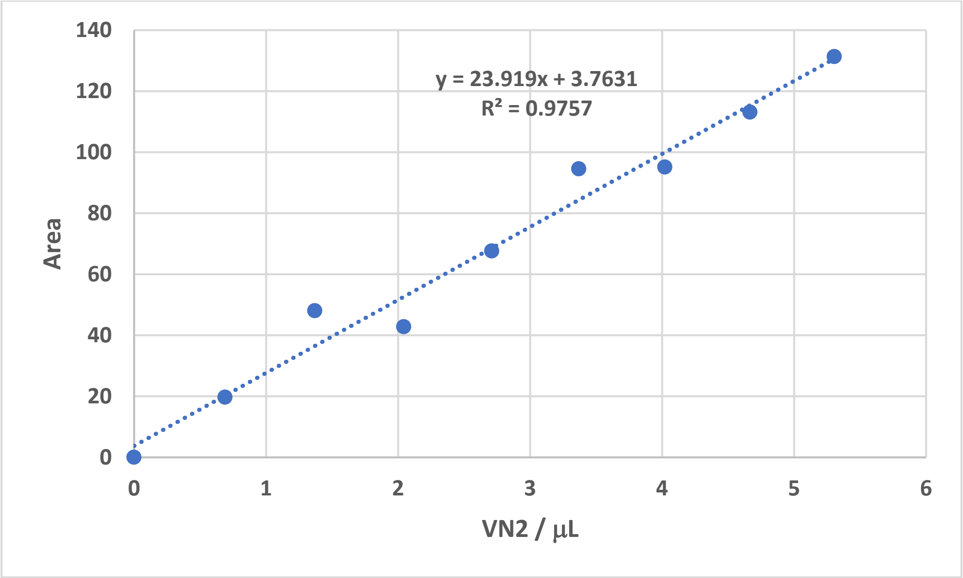

Figure S30. Calibration curve for quantification of N_2 using known mixtures of N_2/N_2O (injection volume, 100 µL). Areas were corrected by subtracting the amount of N_2 from air in the needle determined by the sample containing pure N_2O.

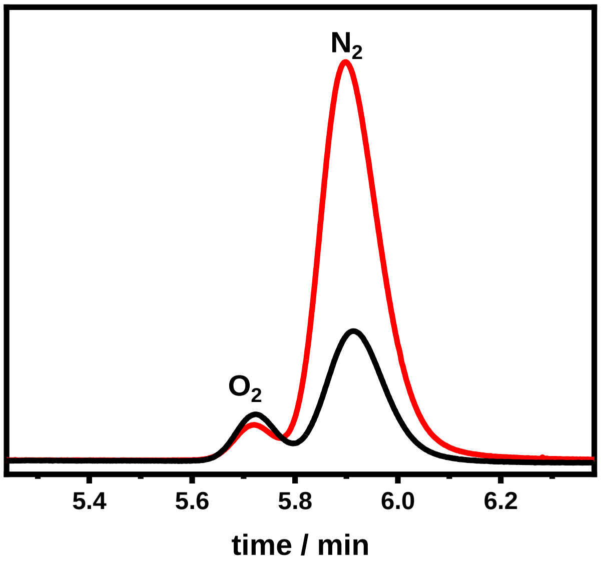

Figure S31. Headspace analysis before (black) and after controlled potential electrolysis (red). The area of O_2 from air remains constant in the chromatogram before and after electrolysis, suggesting there is no adventitious N_2 from air at the end of the experiment.

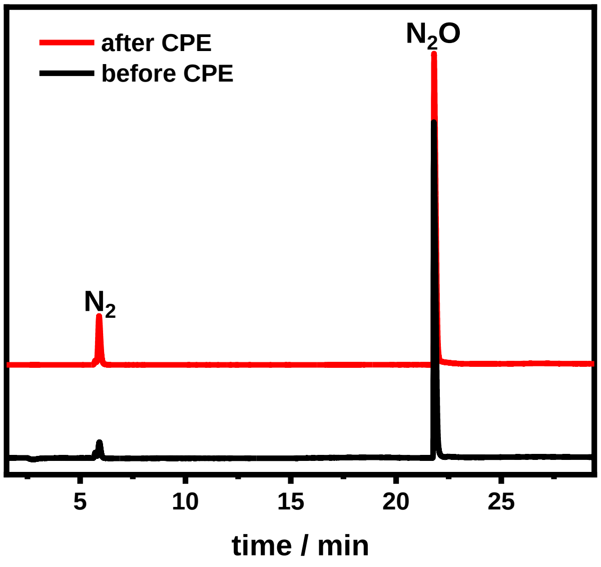

Figure S32. Headspace analysis before (black) and after controlled potential electrolysis (red). Full chromatogram shows that N_2 is the only gaseous product in the reaction.

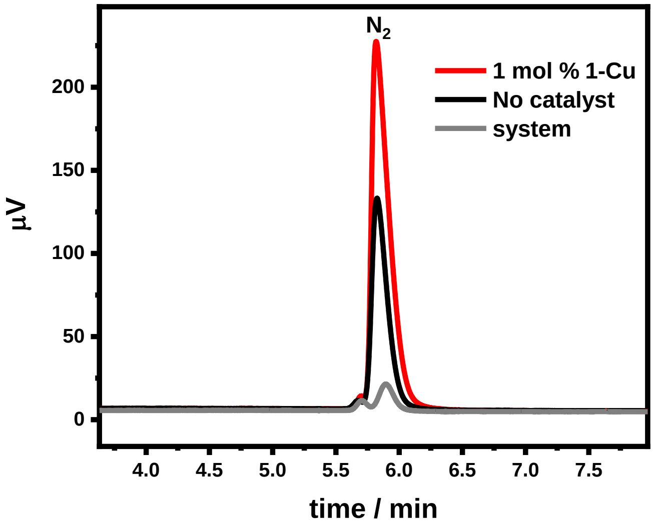

1 mol % 1-Cu	Area	2020.7
	Cal.Curve Vol.	84.32363
	Inj. Vol. (uL)	100
	%N2	84.32363
	VN2 (mL)	31.0311
	mol N2	0.001385
no catalyst	Area	984.8
	Cal.Curve Vol.	41.01496
	Inj. Vol. (uL)	100
	%N2	41.01496
	VN2 (mL)	15.09351
	mol N2	0.000674

Figure S33. Headspace sampling of the reduction of N_2O using 0.1 % Na/Hg as a reducing agent and H_2O as proton source under the presence of 1 mol % loading of 1-Cu (red) and in the absence of catalyst (black) over the course of 16h. Gray trace in chromatogram is the starting amount of N_2 in the system before the start of the experiment.

Controlled Potential Electrolysis (CPE) in the Absence of N_2O

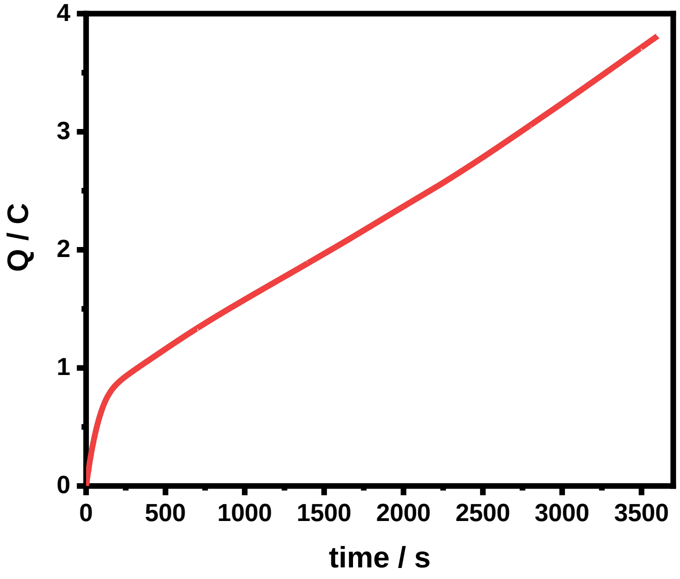

Figure S34. Controlled potential electrolysis at −2.3 V (vs Ag^+/Ag) showing charge passed as a function of time of a MeCN solution containing 1 mM of catalyst (**1-Cu**) and 100 mM H_2O under 1 atm of N_2. RVC working electrode, Ag^+/Ag wire reference electrode, Zn rod counter electrode; MeCN + 0.1 M $N^nBu_4PF_6$ supporting electrolyte.

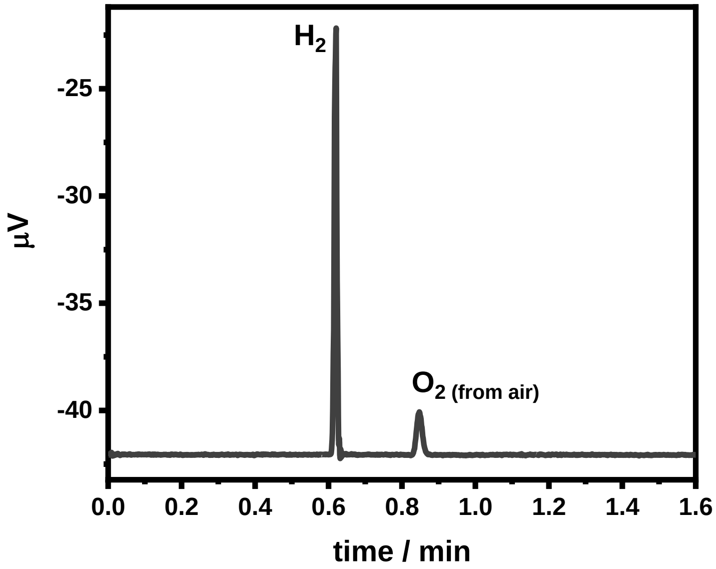

Figure S35. Headspace analysis after controlled potential electrolysis under 1 atm of N_2 in the presence of 100 mM H_2O. TCD GC Chromatogram shows that H_2 evolution in the reaction in the absence of N_2O.

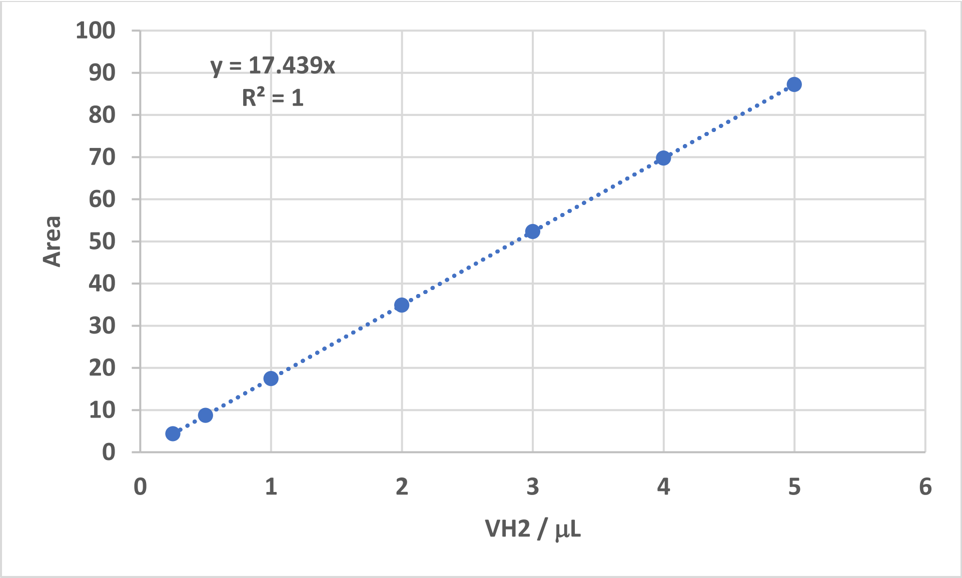

Area	10.33
Cal.Curve Vol. (uL)	0.592350479
Inj. Vol. (uL)	300
%H2	0.19745016
VH2 (mL)	0.017375614
mol H2	7.75697E-07
Q	3.81
Faradaic Efficiency	3.929384035

Injection volume 300 μL; N_2 carrier gas, flow 10 mL/min; Inlet 200 °C; Oven: 35 °C (9 min); Split ratio 10:1; TCD detector 350 °C.

Figure S36. Calibration curve for H_2 quantification in the headspace after controlled potential electrolysis of **1-Cu** (1 mM) under 1 atm of N_2 in the presence of 100 mM H_2O. The amount of H_2 found in the headspace is less than stoichiometric with respect to the catalyst, affording 0.76×10^{-6} mol of H_2 per 4.0×10^{-6} mol of **1-Cu** over 1 h. This indicates that **1-Cu** is not a catalyst for H_2 evolution using H_2O as a substrate at −2.3 V (vs Ag^+/Ag).

UV-Vis Spectroscopy

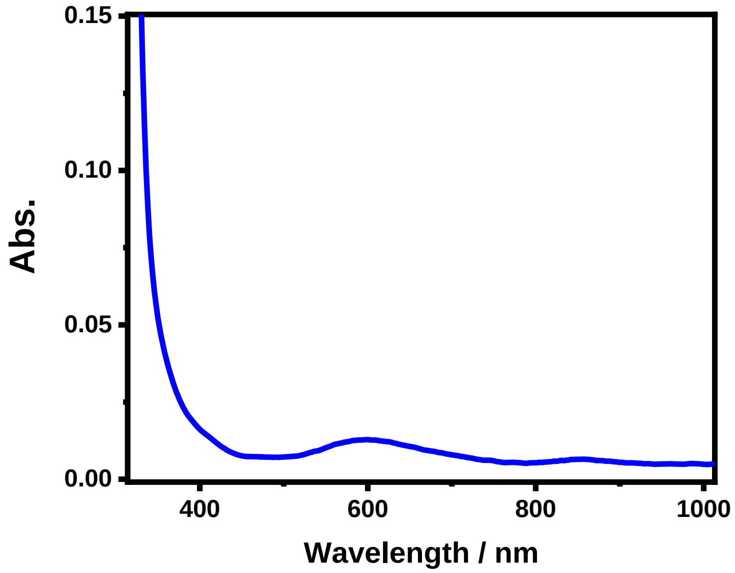

Figure S37. UV-vis spectrum of **1-Cu** (0.5 mM) in MeCN.

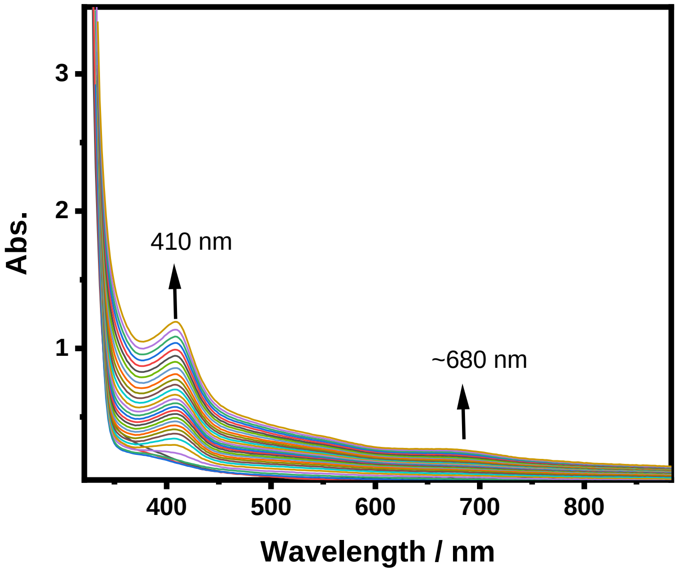

Figure S38. UV-vis spectrum of the reduction of **1-Cu** (0.5 mM) in MeCN in the presence of excess Na/Hg showing the growth of two bands.

EPR spectroscopy

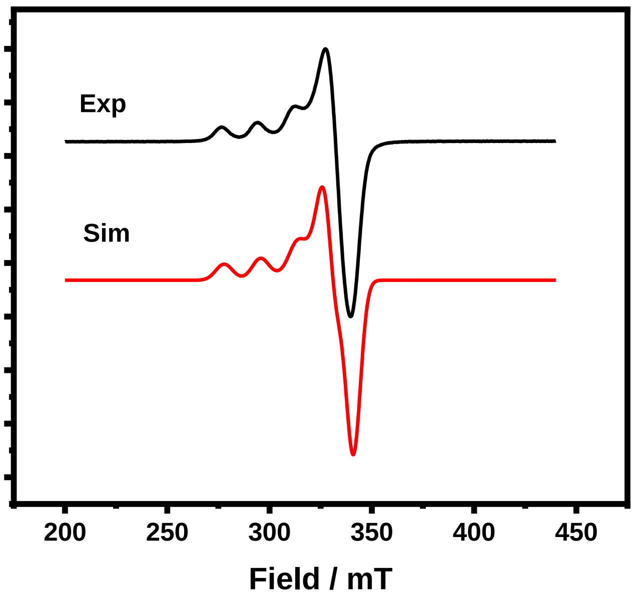

Figure S39. EPR spectrum of **1-Cu** (1mM) in MeCN (15 K). Simulation parameters: g = 2.086, 2.057, 2.261; $A(^{63}Cu)$ = 16, 102, 550 MHz.

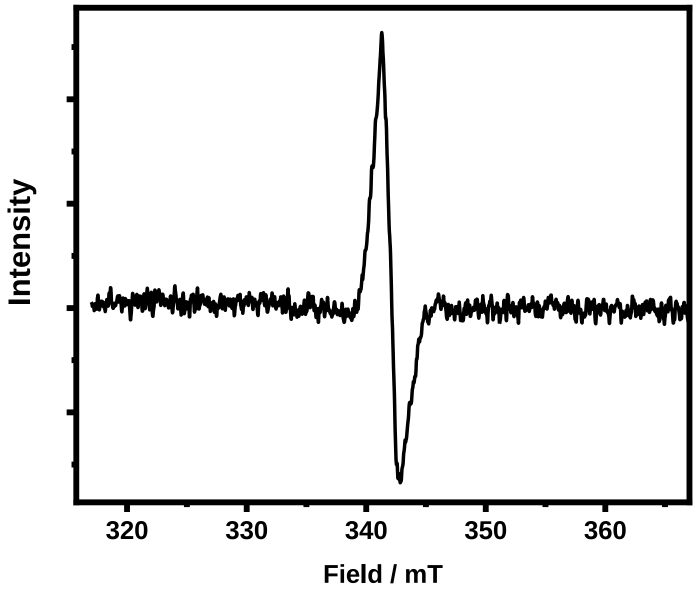

Figure S40. EPR spectrum of the reduction of **1-Zn** using Na/Hg in MeCN (23 K).

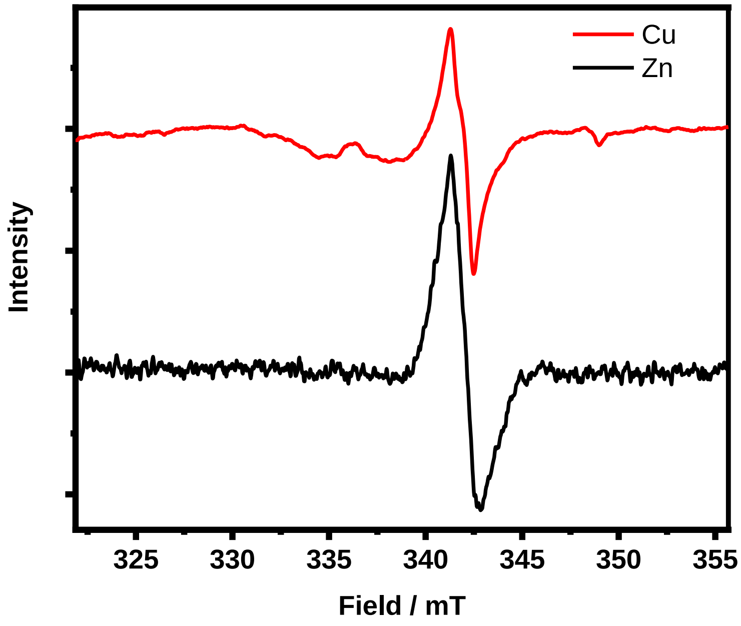

Figure S41. Stacked EPR spectra from the reductions of **1-Cu** and **1-Zn**.

Atom	Alpha	Beta	Gamma	Ax	Ay	Az
0Cu	33.2	25.8	-67.2	-13.80	-5.82	-42.16
1N	-118.1	20.5	131.2	8.02	6.76	6.96
6N	153.2	23.4	-109.0	1.01	0.38	0.29
12N	-41.6	44.8	54.1	6.35	5.36	5.55
17N	-9.0	34.7	-20.5	1.49	0.81	0.70
23N	125.3	17.0	-130.0	1.50	0.94	1.10
28N	126.6	29.7	-104.1	0.56	-0.14	-0.03
34N	151.0	38.0	-147.7	1.91	1.19	1.47
39N	-144.7	5.2	155.0	0.09	-0.22	-0.10
45N	-55.0	29.8	74.8	47.05	-2.97	-3.27
47P	-73.6	21.2	72.0	-3.04	-26.68	-20.14
55P	-34.7	42.8	73.0	3.00	-29.56	-21.39
49H	-18.9	20.3	33.0	-1.98	0.01	2.14
51H	-53.6	31.4	71.5	-21.06	-35.75	-9.97
53H	4.9	30.9	65.7	-0.59	1.65	4.26

Table S1. Output from calculated A values (MHz) of the EPR of the 2e$^-$ reduced (ligand radical) model complex. Euler angles are reported in z-y-z convention. See DFT section below for computational details.

Single Crystal X-ray Diffraction (SXRD)

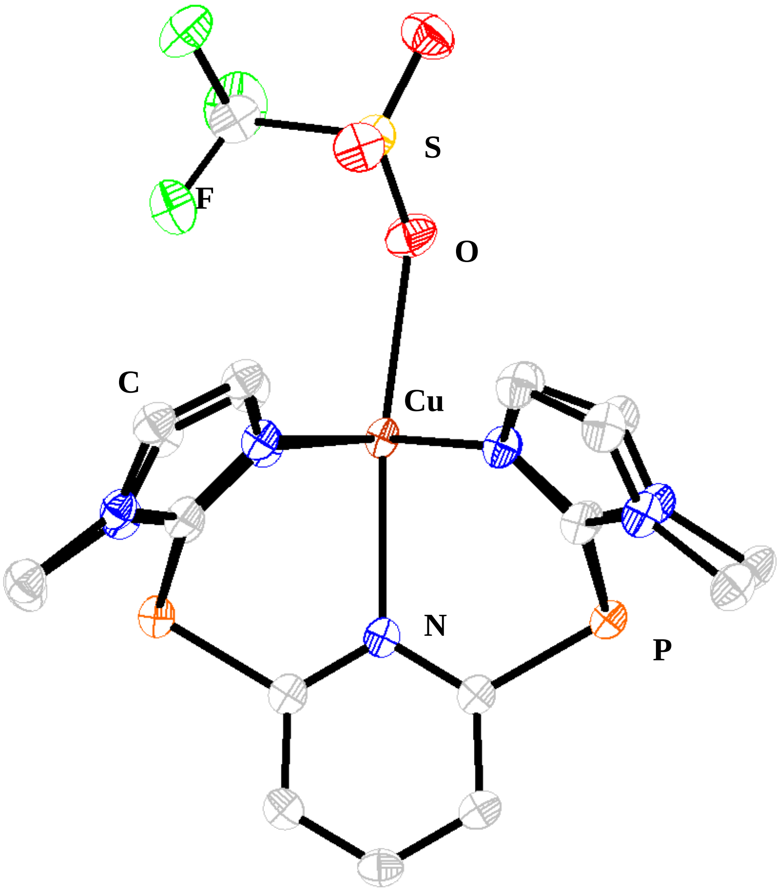

Cu-N$_{Im}$	2.048(2), 2.013(2), 2.028(2), 1.997(2) Å
Cu-N$_{Py}$	2.380(2) Å
Cu-OTf	2.06(1) Å, 2.206(4)
<C-P-C>	98.4(1)°

Figure S42. Solid state structure of **1-Cu** and select bond distances and angles in the table below. Ellipsoids are set to 50% probability and hydrogen atoms, triflate counterion and acetonitrile solvent molecule have been omitted for clarity. C shown in gray, N in blue, O in red, F in green, P in orange, S in yellow, and Cu in brown.

Table S2. Crystal data and structure refinement for **1-Cu**.

Empirical formula	$C_{25}H_{26}CuF_6N_{10}O_6P_2S_2$	
Formula weight	866.16	
Temperature	100(2) K	
Crystal system	monoclinic	
Space group	$P2_1/n$	
Unit cell dimensions	a = 13.1535(8) Å	α = 90°
	b = 13.4486(8) Å	β = 108.9070(10)°
	c = 20.1548(13) Å	γ = 90°
Volume	3372.9(4) Å^3	
Z	4	
Pcalcg	1.706 cm^3	
M	0.226 mm^{-1}	
F(000)	1756.0	
Crystal size	0.08 × 0.07 × 0.07 mm^3	
Radiation	synchrotron (λ = 0.41328)	
2Θ range for data collection	1.906 to 32.054°	
Index ranges	-17 ≤ h ≤ 17, -16 ≤ k ≤ 17, -26 ≤ l ≤ 26	
Reflections collected	101485	
Independent reflections	7936 [Rint = 0.0842, Rsigma = 0.0518]	
Data/restraints/parameters	7936/0/474	
Goodness-of-fit on F^2	1.037	
Final R indexes [I>=2σ (I)]	R1 = 0.0402, wR2 = 0.0997	
Final R indexes [all data]	R1 = 0.0512, wR2 = 0.1067	
Largest diff. peak/hole	0.57/-0.82 e Å^{-3}	

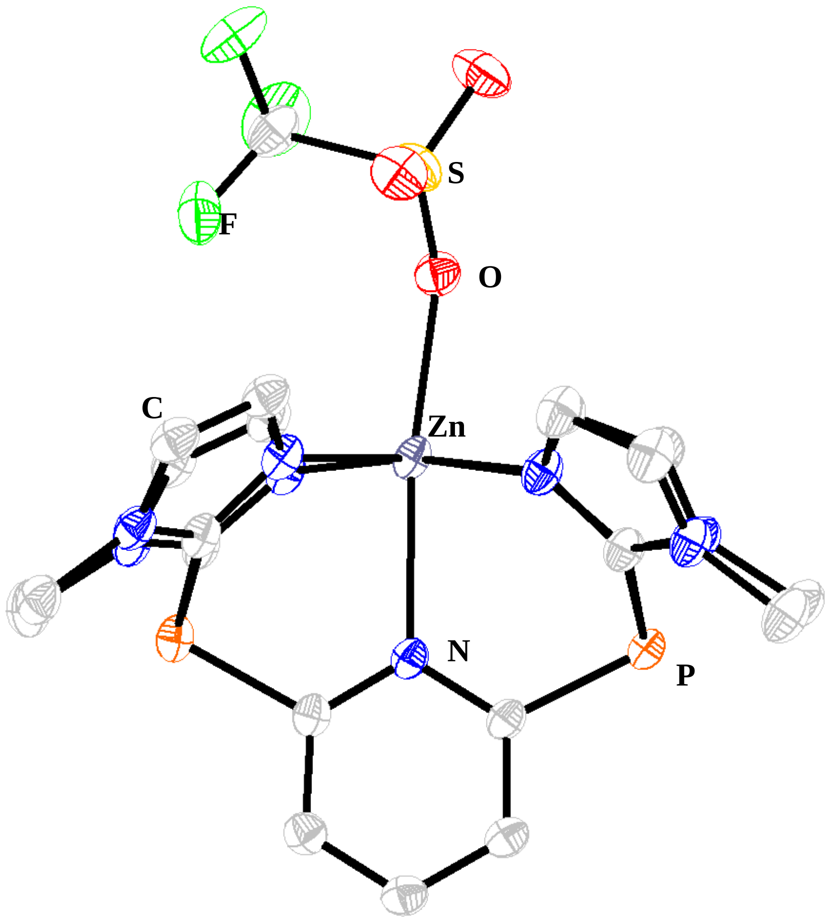

Zn-N$_{Im}$	2.082(1), 2.103(1), 2.123(1), 2.163(1) Å
Zn-N$_{Py}$	2.380(2) Å
Zn-OTf	2.06(1) Å, 2.206(4)
<C-P-C>	98.9(8)°

Figure S43. Solid state structure of **1-Zn** and select bond distances and angles in the table below. Ellipsoids are set to 50% probability and hydrogen atoms have been omitted for clarity. C shown in gray, N in blue, O in red, F in green, P in orange, S in yellow, and Zn in silver.

Table S3. Crystal data and structure refinement for **1-Zn**.

Empirical formula	C25H26F6N10O6P2S2Zn	
Formula weight	867.99	
Temperature	100(2) K	
Crystal system	monoclinic	
Space group	P21/c	
Unit cell dimensions	a = 13.2291(7) Å	α = 90°
	b = 13.4502(7) Å	β = 108.6320(10)°
	c = 20.3253(11) Å	γ = 90°
Volume	3427.0(3) Å^3	
Z	4	
Pcalcg	1.682 cm^3	
μ	0.241 mm^{-1}	
F(000)	1760.0	
Crystal size	0.08 × 0.07 × 0.07 mm^3	
Radiation	synchrotron (λ = 0.41328)	
2Θ range for data collection	1.89 to 40.894°	
Index ranges	-22 ≤ h ≤ 22, -22 ≤ k ≤ 22, -34 ≤ l ≤ 34	
Reflections collected	231303	
Independent reflections	16974 [Rint = 0.0479, Rsigma = 0.0230]	
Data/restraints/parameters	16974/0/528	
Goodness-of-fit on F^2	1.120	
Final R indexes [I>=2σ (I)]	R1 = 0.0552, wR2 = 0.1532	
Final R indexes [all data]	R1 = 0.0666, wR2 = 0.1592	
Largest diff. peak/hole	1.33/-1.93 e Å^{-3}	

Density Functional Theory (DFT)

Density functional theory calculations were performed in the ORCA 5.0.2 software suite with the TPSSh functional, the RI approximation, a D3 dispersion correction with Becke-Johnson damping, and a conductor-like polarizable continuum model with the parameters of acetonitrile.[4-8] The basis sets of Weigand and Ahlrichs were used;[9-10] copper was given the def2-TZVPP functional, all atoms bound to copper were given def2-TZVP, and all other atoms were given def2-TZVP(-f). Optimized structures were confirmed by the absence of negative eigenvalues of the Hessian. Entropy and Gibb's free entropy were determined at 296 K and 1 M using the quasi-QRRHO model for Grimme and coworkers.[11] For EPR calculations on the reduced ligand radical, copper was given def2-QZVP, atoms bound to copper, phosphorous, and the 3-positions of the imidazole arms were given def2-TZVPP. Hyperfine couplings were calculated with isotropic and dipolar contributions on all copper, nitrogen, and phosphorus, and pyridyl hydrogen nuclei and orbital contributions on copper.

	Electronic Energy (kcal/mol)	Enthalpy (kcal/mol)	Gibb's Free Entropy (kcal/mol)
Cu(II)OTf[a]	0	0	0
Cu(II) + $^{-}$OTf	11.1	10.0	−0.6[b]
Cu(I), 4-Coordinate[a]	0	0	0
Cu(I)_T, 3-Coordinate	4.7	4.9	5.1
Cu(I), Reduced Ligand, 4-Coordinate[a,c]	0	0	0
Cu(I), Reduced Ligand, 3-Coordinate	0.9	1.1	1.7

Table S4. Summary of calculated energies from DFT calculations. The free energy of disproportionation of two Cu(I) species into a Cu(II) species and Cu(I), reduced ligand species is calculated to be 31.4 kcal/mol, compared to 31.1 kcal/mol from the electrochemical data. [a]Energy reference. [b]At standard conditions with all species at 1 M. [c]EPR parameters were calculated for this species.

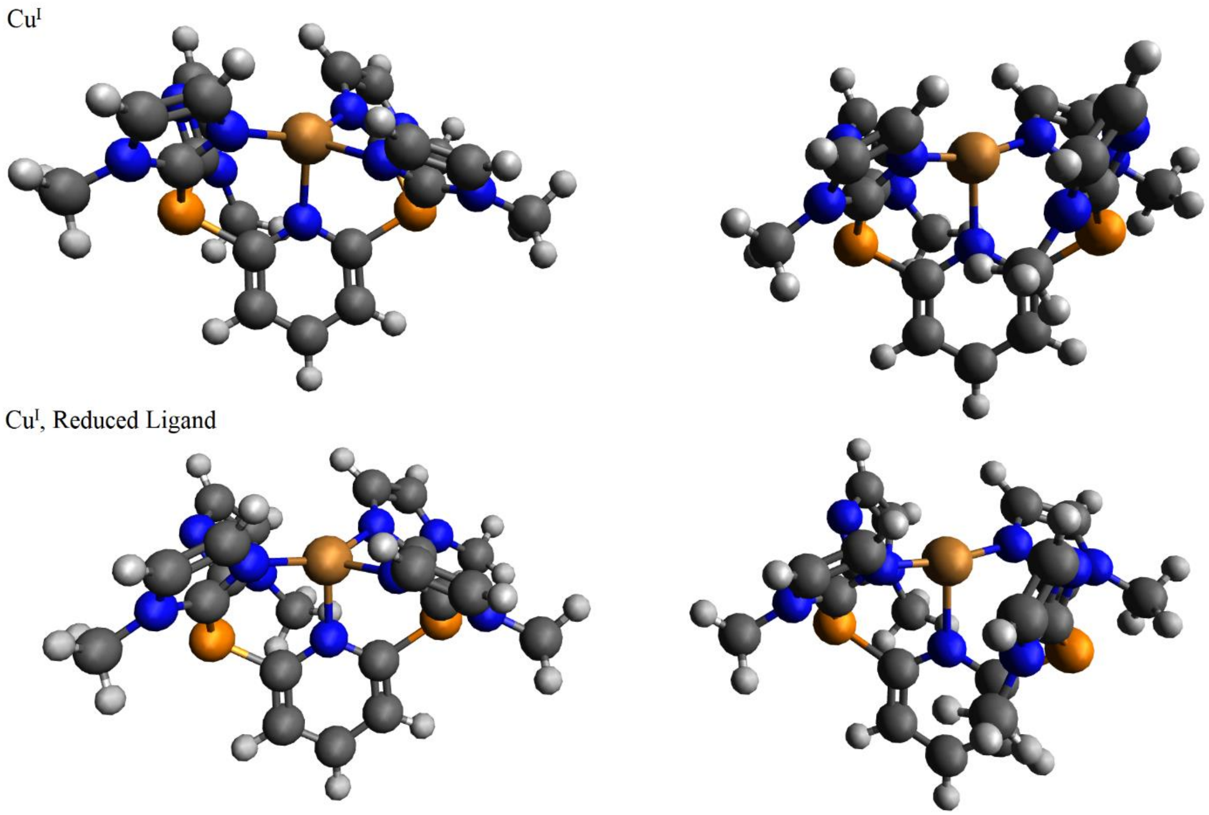

Figure S44. 4-Coordinate (Left) and 3-Coordinate (right) optimized structures for the Cu(I) (top) and Cu(I), reduced ligand species (bottom). Other permutations of two-arms unbound are also likely accessible. XYZ coordinates are available in the online supporting information.

REFERENCES

1. Frogneux, X.; von Wolff, N.; Thuéry, P.; Lefèvre, G.; Cantat, T., CO2 Conversion into Esters by Fluoride-Mediated Carboxylation of Organosilanes and Halide Derivatives. *Chem. Eur. J.* **2016,** *22* (9), 2930-2934.
2. Stoll, S.; Schweiger, A., EasySpin, a comprehensive software package for spectral simulation. *J. Magn. Reson.* **2006,** (178), 42-55.
3. Deeba, R.; Molton, F.; Chardon-Noblat, S.; Costentin, C., Effective Homogeneous Catalysis of Electrochemical Reduction of Nitrous Oxide to Dinitrogen at Rhenium Carbonyl Catalysts. *ACS Catal.* **2021,** *11* (10), 6099-6103.
4. Neese, F. W., F.; Becker, U.; Riplinger, C. , The ORCA Quantum Chemistry Program Package. *J. Chem. Phys.* **2020,** *152* (22), 224108-224118.
5. Tao, J. P., J. P.; Staroverov, V. N.; Scuseria, G. E., Climbing the Density Functional Ladder: Nonempirical Meta - Generalized Gradient Approximation Designed for Molecules and Solids. *Phys. Rev. Lett. 91* (14), 146401-146404.
6. Grimme, S. A., J.; Ehrlich, S.; Krieg, H. J., A Consistent and Accurate Ab Initio Parametrization of Density Functional Dispersion Correction (DFT-D) for the 94 Elements H-Pu. *J. Chem. Phys.* **2010,** *132* (15), 154104-154119.
7. Grimme, S. E., S.; Goerigk, L. J., Effect of the Damping Function in Dispersion Corrected Density Functional Theory. *J. Comput. Chem.* **2011,** *32* (7), 1456–1465.
8. York, D. M. K., M., A Smooth Solvation Potential Based on the Conductor-Like Screening Model. *J. Phys. Chem. A* **1999,** *103* (50), 11060–11079. .
9. Weigend, F., Accurate Coulomb-Fitting Basis Sets for H to Rn. *Phys. Chem. Chem. Phys.* **2006,** *8* (9), 1057–1065.
10. Weigend, F. A., R., Balanced Basis Sets of Split Valence, Triple Zeta Valence and Quadruple Zeta Valence Quality for H to Rn: Design and Assessment of Accuracy. *Phys. Chem. Chem. Phys.* **2005,** *7* (18), 3297–3305.
11. Grimme, S., Supramolecular Binding Thermodynamics by Dispersion-Corrected Density Functional Theory. *Chem. Eur. J.* **2012,** *18* (32), 9955–9964.
